# Recent Advances in Materials and Testing Methodologies for Soft Body Armor

**DOI:** 10.3390/polym18131628

**Published:** 2026-06-30

**Authors:** Rahul Chamola, Tabrej Khan, Tamer A. Sebaey, Subhankar Das, Harri Junaedi, Manjeet Singh Goyat

**Affiliations:** 1 Mechanical Engineering Cluster, School of Advanced Engineering, University of Petroleum and Energy Studies, Dehradun 248007, Uttarakhand, India; rahul.chamola@ddn.upes.ac.in; 2 Engineering Management Department, College of Engineering, Prince Sultan University, P.O. Box 66833, Riyadh 11586, Saudi Arabia; tkhan@psu.edu.sa (T.K.); tsebaey@psu.edu.sa (T.A.S.); hlukman@psu.edu.sa (H.J.); 3 Mechanical Design and Production Department, Faculty of Engineering, Zagazig University, Zagazig 44519, Sharkia, Egypt; 4 Cluster of Applied Science, School of Advanced Engineering, University of Petroleum and Energy Studies, Dehradun 248007, Uttarakhand, India

**Keywords:** soft body armor, ballistic impact behavior, fabric architecture, shear thickening fluids, energy absorption mechanisms

## Abstract

The ballistic impact behavior of soft body armor is governed by complex interactions between material architecture and projectile characteristics. This review provides a critical overview of the evolution of textile and composite-based armor materials developed for ballistic protection. Emphasis is placed on experimental and analytical methodologies used to elucidate impact energy dissipation, deformation mechanisms, and failure modes. Key material-related parameters influencing ballistic performance including areal density, weave architecture, yarn crimp, twist, and thread density are systematically discussed, along with assembly variables such as ply orientation, layer number, and hybrid configurations. In parallel, the influence of projectile mass, velocity, and geometry on impact resistance is examined. The review also summarizes internationally adopted ballistic and stab-resistance standards employed for soft armor evaluation. Various assessment techniques, including yarn–yarn friction analysis, puncture resistance testing, ballistic limit velocity determination, and back-face signature measurement, are critically reviewed. Strategies aimed at enhancing impact performance, such as rubber or latex impregnation, fiber surface modification, and the incorporation of shear thickening fluids, are comprehensively discussed. Attention is given to shear thickening fluids due to their significant role in improving energy absorption and flexibility. The fundamental mechanisms governing shear thickening behavior and the parameters affecting their performance are analyzed. Overall, this review highlights emerging material design strategies and performance optimization approaches for next-generation soft body armor systems.

## 1. Introduction

The development of counterattack and protection weapons against external threats has continued throughout human history. Emerging conflicts have driven humans to develop body armor to safeguard themselves against the effects of weapons. The armor system was introduced to provide the utmost protection against threats like stabbing, explosions, and high-impact penetration. Consequently, primitive materials such as leather, wood, and steel became popular for crafting both weapons and protective shields in the early stages [1,2]. The armor is classified as either hard (rigid) or soft, depending on the materials used to protect against various threats. The hard body comprises a metal, ceramic, and polyethylene layer, used mainly for high protection against high-velocity impact loads [3]. On the contrary, soft body armor is constructed from several layers (generally 20–50) of high-performance fabric to achieve low to moderate ballistic protection [4]. Despite offering high-level protection against ballistic impact loads, the demand for hard body armor has declined for threats from low-velocity impact over the last few decades due to its inflexibility and unfavorable weight-to-strength ratio. In contrast, high-performance synthetic fibers were found to provide excellent strength and modulus, as well as enhanced chemical resistance, compared to hard body armor [5].

Over time, the popularity of hard and soft body armor has increased with the rise of global conflicts. Soft body armor provides high flexibility and comfort, especially for lower National Institute of Justice (NIJ) threat levels. In contrast, hard body armor is typically preferred for protection against high-velocity impacts. However, the use of these armors is often limited due to excessive weight and discomfort to the wearer. Therefore, various fabrics and laminates composed of traditional fibers, such as nylon and Kevlar, were introduced in the late 1939s for ballistic protection systems [6]. Until the 1970s, nylon was accepted as a standard fiber and a ballistic material; however, other high-performance fabrics, such as para-aramid and ultra-high-molecular-weight polyethylene (UHMWPE), were introduced by DuPont in 1965 to further advance the field [7]. Para-aramid (Kevlar and Twaron) and UHMWPE (Spectra and Dyneema) are two well-known high-performance fabrics that researchers have investigated for high-impact-resistant applications [7,8]. Early researchers achieved protection by adding up to 40 fabric layers to meet body armor safety standards; however, this created a bulky design and reduced the wearer’s flexibility [9]. Researchers faced the primary challenge of minimizing weight and enhancing the flexibility of body armor without compromising its strength. They resolved these issues in the early 2000s by introducing shear-thickening fluids (STFs) [10].

STF is a type of intelligent ballistic-resistant material that is composed of solid particles suspended in a dispersion medium [11]. STF exhibits non-Newtonian characteristics, where its viscosity abruptly changes with an increased shear rate. The fluid exhibits reversible behavior, transitioning from a liquid to a solid phase at high shear rates, making it a potential material for absorbing impact energy [12,13,14]. Hence, this feature of STFs is used to impregnate high-performance fabrics, intended to boost the impact resistance of fabric under high-impact loading. Maximum attention is being given to soft body armor instead of hard armor due to its advantages for the wearer, as evidenced by current research trends in Section 1.1.

The novelty of this review is reflected in its combined assessment of both the material level and the projectile level governing ballistic performance, and at the same time integrating these fundamentals into the contemporary improvement techniques. Unlike previously reported reviews that focus narrowly on fabrics or testing standards, the current work correlates the fabric architecture, assembly parameters, projectile attributes, and advanced reinforcement methods such as shear thickening fluids, latex coatings, and fiber modifications. Additionally, the review provides an in-depth analysis of emerging evaluation techniques, namely, yarn friction, puncture resistance, ballistic limit velocity, and back-face signature. Thus, this review provides a comprehensive and layered analysis that helps new researchers to understand the design and development of next-generation soft body armor.

### 1.1. Bibliometric Analysis

**Figure 1:**
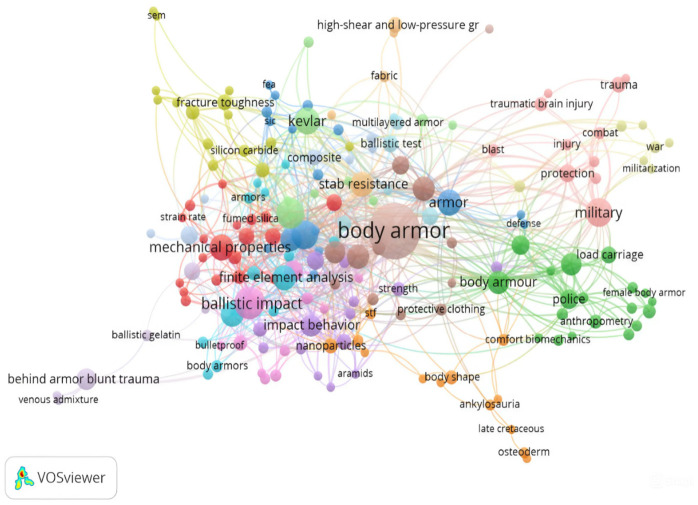
Network visualization map of keywords used by worldwide authors in their documents for body armor research from 2011–2025: (Web of Science data, assessed on 6 December 2025).

The bibliometric analysis was conducted using the Web of Science database by retrieving publications from last 15 years with the keywords “body armor” and “body armour”, which yielded 1316 and 1699 research articles, respectively. However, only 259 of these specifically focus on “soft body armor,” indicating a novelty and a significant scope of research on this topic. The co-occurrence of keywords used in research by worldwide scholars and scientists is visualized in Figure 1. The node size represents the frequency of each keyword. The large node size represents the most frequent keywords in the retrieved literature. It is clear from Figure 1 that keywords such as body armor, ballistic impact, stab resistance, Kevlar, fumed silica, finite element analysis, and impact behavior have been the focus of previous studies.

**Figure 2:**
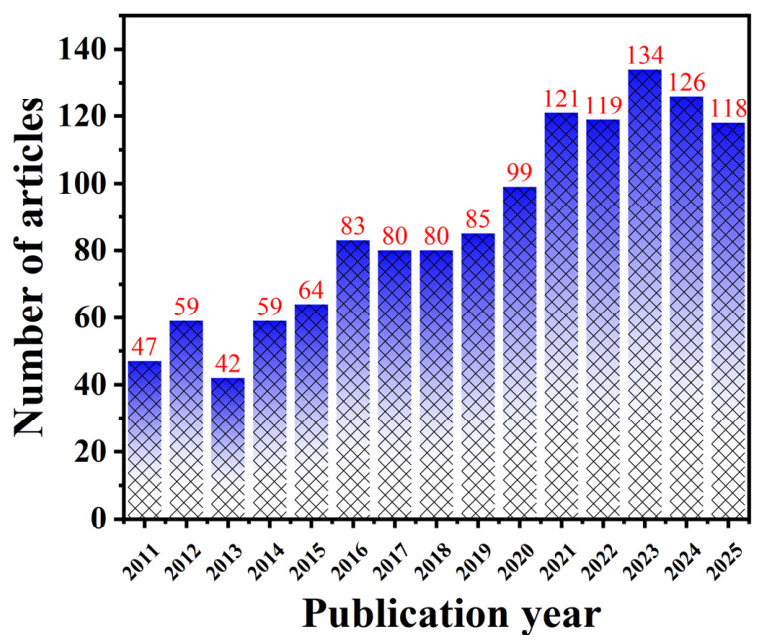
Number of articles published from 2011 to 2025 (Web of Science data, accessed on 6 December 2025).

**Figure 3:**
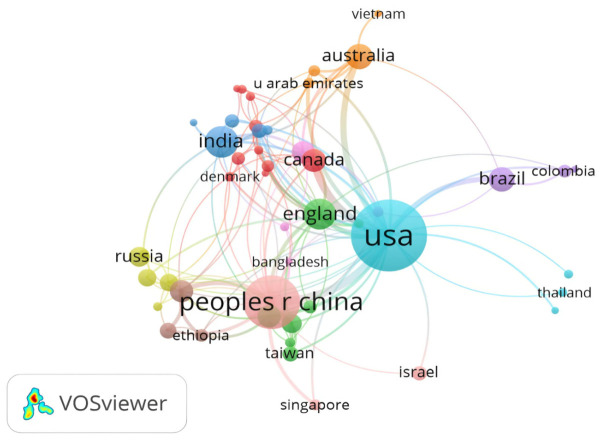
Network visualization map of research outcomes of top active countries with at least 3 articles on body armor (Web of Science data, assessed on 6 December 2025).

The bar diagram (Figure 2) represents the research outcomes for both soft and hard body armor from 2011 to 2025 A total of 1316 articles were published on body armor-related keywords. Researchers from countries such as the United States, China, the United Kingdom, and India have emerged as the leading contributors to body armor research, dominating the field’s publication landscape. A bibliometric analysis (Figure 3) using the Web of Science database reveals that these countries consistently published several papers from 2011 to 2025 (as of 6 December 2025). The collaboration patterns depicted in Figure 3 reflect co-authorship links rather than subjective interpretation. A relatively stronger connection between China and Russia was found compared to Russia and the USA, which may be attributed to evolving geopolitical alignments, regional research cooperation, and defense-related collaboration policies.

For this analysis, only countries with at least three publications during this period were considered. The pictographic representation reflects global research trends in body armor technologies.

### 1.2. Evolution of Body Armor Materials

Over the centuries, humans have adopted various tactics to protect themselves from the environment, animals, and enemies. Depending on the threats, protection was ensured in multiple ways, such as seeking safe shelters to avoid proximal threats, fleeing for survival, and using protective shields and weapons to confront situations directly. Among all the tactics humans used, protective shields/armor were popularized as a personal protection system [1]. In the advancement of human history, the Persians and Greeks acquired significant expertise in developing more sophisticated weapons, respectively, around 600 BC [15]. The Persians used large bronze plates mounted on leather harnesses, while the Greeks used iron plates mounted on leather harnesses. Later, steel-plated armor was introduced during the medieval period of European history to achieve greater flexibility in combat. The steel-plated armor disappeared from infantry after the 18th century due to its ineffectiveness and weight against contemporary weapons. Besides metal protection systems, Chinese and Mongolian warriors used fabric armor, such as leather and animal skin, from the 11th to the 13th century CE [16]. Moreover, quilted linen coats were used in northern India until the 19th century. Despite the advancement in protection systems, the devastating casualties faced by troops during World War I were a result of advanced weapons like machine guns, snipers, and fragments. Therefore, Coates and Bayers [1] conducted a systematic study to investigate the impact of firearms on various parts of the body. They found that the lower and upper limbs were most affected, at 39% and 22%, respectively, followed by the chest at 16% and the head and neck at 12% [17,18].

During the later part of the Korean War, the US introduced the M-1952 (a model code name for a nylon-based body armor) [19]. The flexible vest weighed 3.6 kg and consisted of 12 layers of laminated nylon, offering improved ballistic protection against shell fragments. Subsequently, they introduced M-1969 (a 15-layered nylon-based body armor) to attain the high protection efficiency of the existing M-1952. The performance of nylon-based flexible vests has set a new benchmark for other fabrics to explore their potential to resist ballistic threats [20]. Therefore, researchers have begun exploring other synthetic fibers in their quest for improved ballistic resistance, reduced weight, and high tensile strength against various threats, which are elaborated upon in Section 1.3. The global synthetic fiber market was valued at approximately 98.89 billion USD in 2025 and is projected to reach 176.88 billion USD by 2034, as per the report of Fortune Business Insights. The Asia-Pacific held the largest share, accounting for 65.10% of total technical textile market, with the leading countries being China, India, and Japan.

This review aims to provide an in-depth understanding of findings from worldwide researchers to design a soft body armor that can be effectively integrated into current scenarios. The article examines the historical evolution of body armor from ancient times, highlighting its benefits and limitations. The year-by-year development of armor materials and their performance under both low- and high-velocity impacts is discussed. Furthermore, parameters influencing impact energy absorption, including fiber properties, yarn parameters, fabric layers, ply arrangement, hybridization, and fabric structure, are discussed. Additionally, the review examines the impact of projectile geometry on the performance of fabric materials, providing a rigorous analysis of various testing standards. The underlying mechanism of STFs, as well as the influence of particle shape and size, volume fraction, hardness, roughness, and carrier medium, is explained. Emphasis is placed on the potential use of STF-impregnated naturally driven fabric and its characterization. It is believed that the current technology used in STF-impregnated synthetic fabric composites has the potential to be leveraged in the fabrication of natural fiber composites designed for ballistic applications. Hence, the article also provides the reader with deeper insight into STF-impregnated natural fiber composites and their applications in the design of protective systems.

### 1.3. Advancement in STF

**Figure 4:**
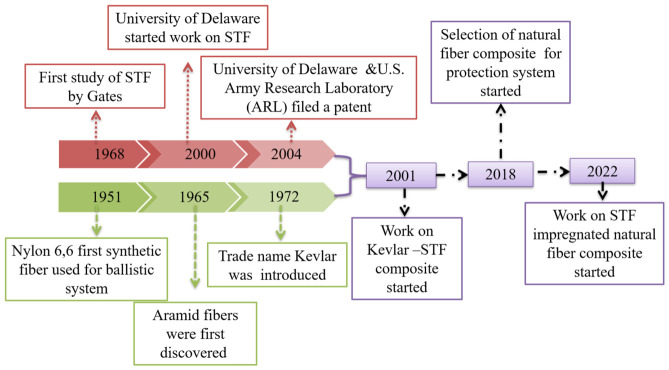
Year-by-year evolution in designing soft body armor (Refs. [2,6,7], assessed on 16 February 2025).

Further, the work was extended to STF-impregnated high-performance fabric for the development of lightweight, flexible armor. However, their use is limited due to key issues such as high cost, low STF retention, and non-biodegradability [21]. Therefore, researchers have made efforts by hybridizing traditional fibers with naturally derived fibers [1,22,23,24,25,26]. Recently, STF-impregnated jute fabrics have been evaluated for their ballistic performance and puncture resistance. The findings of this research confirmed the potential of jute fabrics for developing sustainable protective armors and gears in the future [19]. Figure 4 exhibits the chronological advancement of body armor materials and technologies, outlining two separate lines of development that converge over time [2,6,7]. The lower timeline illustrates the evolution of protective fibers, starting with nylon 6,6 in 1951 as the first synthetic ballistic material, followed by the discovery of aramid fibers in 1965 and the introduction of Kevlar in 1972, which enhanced the protective performance of fabric. The upper timeline shows the development of STF, starting from 1968 by Gates [8], followed by major contributions from the University of Delaware in 2000 and eventually patent efforts with the U.S. Army Research Laboratory in 2004. Around 2001, both lines converge with the development of Kevlar–STF composites, marking the transition to hybrid systems. In recent years, research has further progressed toward sustainable solutions, focusing on natural fiber-based composites to explore their potential for environmentally friendly body armor practices [27,28], which are discussed in further sections.

### 1.4. Protection Against Different Types of Threats

Different kinds of body armor have been designed to safeguard against handguns, rifles, automatic weapons, sniper rifles, fragments, and stabbing attacks. Various types of body armor are classified according to their resistance to penetration by different bullets and calibers, as per NIJ-0101.06 [29]. Depending on the threat level, troops can choose between soft and semi-rigid (a combination of fabric and metal) body armor. Fragments from an explosive can be harmful to humans, including sharp, small metal pieces that body armor can easily tackle [30]. Several sharp, pointed stabbing tools, including domestic knives, utility knives, and spiking objects, have been in use by humans for single or multiple cutting, slashing, and piercing.

**Figure 5:**
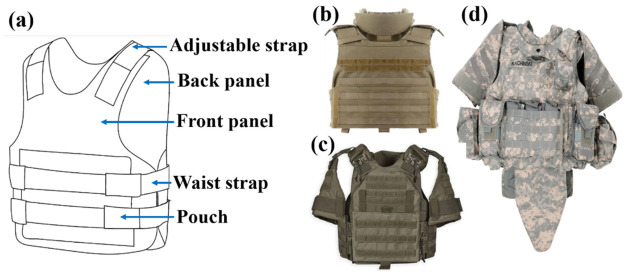
Soft body armor: (**a**) nomenclature, (**b**) neck protection, (**c**) shoulder protection, and (**d**) groin protection.

Ballistic armor is designed to protect the wearer against different threats. The armor must be lightweight and flexible for modern warfare [31]. Fibrous body armor replaces hard body armor to achieve the highest comfort for the wearer without compromising the impact resistance performance [32]. Nowadays, various technical textiles, such as Kevlar and UHMWPE fibers, are suitable for developing soft body armor. Modern armor also features cooling channels embedded in its structure to improve air circulation. It features attachment points for various components, including protection for the neck, shoulders, and upper and lower limbs [33]. Figure 5 provides a detailed illustration of armor made from synthetic fibers.

Furthermore, the investigation began by analyzing the impact of energy-absorption phenomena in soft body armor through wave propagation in various high-performance fabrics. Section 2 highlights the key investigation reported in previous studies.

## 2. Assessment on Impact Energy Absorption

**Figure 6:**
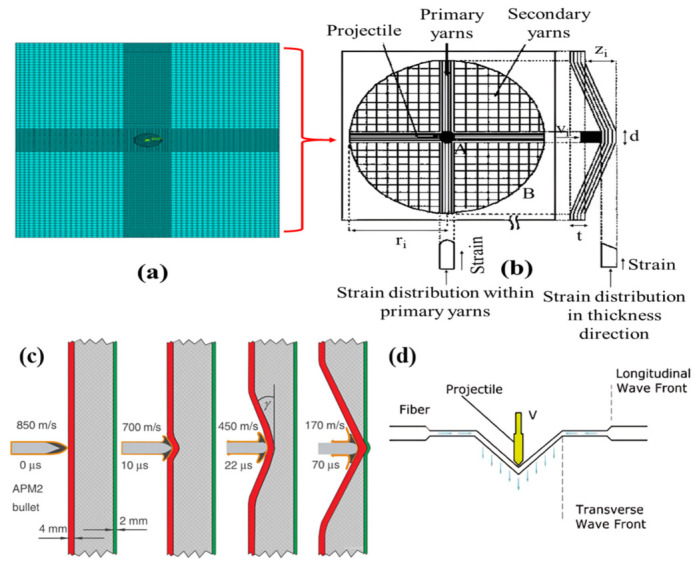
Wave propagation phenomenon in fabric: (**a**) fabric impacted area (reprinted from [9], MDPI, 2024), (**b**) cone formation when hit by a projectile (reprinted with permission from [34], Elsevier, 2006), (**c**) piercing in a lightweight composite (reprinted with permission from [2], Elsevier, 2019), (**d**) transverse deflection in fiber (adapted from [35], MDPI, 2021).

The behavior of high-performance fabric under impact loads can be analyzed by examining the propagation of impact waves and their impact energy absorption performance. This section highlights the different impact waves generated during impact and their absorption by fabric materials. Yarns are continuous strands of fibers used to develop fabric mainly through weaving, knitting, or braiding processes. In heterogeneous and anisotropic materials under impact load, stress waves and deformation spread through the impacted region at defined velocities, resulting in shearing distortion and longitudinal dilation of the material [2]. The wave-propagation phenomenon in a fabric is illustrated in Figure 6a,b [9,34]. Schematic views of the stress distribution on the yarn in the fabric passing through the impact region are presented in Figure 6a. In contrast, Figure 6b exhibits the deformation and cone formation in the fabric during ballistic impact. In Figure 6b, the diameter of the projectile is denoted by d, the radius of the surface of the cone by r_i_, and the distance travelled by the projectile by z_i_ for the investigation of wave propagation in fabric. During ballistic impact, a longitudinal stress wave travels through the yarn in the plane of the fabric, and the transverse wavefront develops a bulging effect, usually in the shape of a semi-circle in the fabric, which helps in the distribution of impact loads.

The wave propagation during high-impact load is also visible in hard armor (light-weight composite) panels, as showcased in Figure 6c,d [2,35]. The piercing in a lightweight composite made up of a 5–10 mm ceramic layer followed by an ultra-hard surface and 2 mm thick fibrous material through the APM2 bullet at 850 m/s is displayed in Figure 6c [2]. It is evident from Figure 6c that the speed of the bullet diminishes as it travels through the composites. As a result, the bullet’s shape was also deformed. Due to high impact, ceramic (hard) surfaces are penetrated, and ultimately, impact waves are generated in the fibrous material and stopped. The transverse defection in the fiber is displayed in Figure 6d. At the site of impact, the yarn in the fibrous material exhibits a minor deformation. With time, the strain wave (longitudinal wave) moves to nearby positions along the yarn axis as the strain progresses at the original impact point and continues to build up.

During ballistic impact, the transverse displacement of primary yarns induces a longitudinal stress wave in the secondary yarns. Furthermore, the transverse deflection attains its highest point when the major yarns fail. Studies indicate that the majority of the impact energy is dissipated by the principal yarns through both kinetic and strain energy. However, secondary yarns contribute little to energy absorption due to the built-in right-angle yarn architecture of plain weave [36]. The longitudinal wave motion occurs at sonic speed, while transverse waves move at a significantly slower pace in the fabric due to yarn defects in the direction of impact. The longitudinal velocity can be expressed with Equation (1) [37].

(1) $$C=\sqrt{\frac{E}{\rho }}$$

where *C* is the velocity in the lengthwise direction, *E* is the tensile modulus, and ρ is the fiber density.

The transverse wave travels at a much lower speed than longitudinal waves, and the speed of the transverse wave is given by Equation (2) [38].

(2) $$u=C\sqrt{\frac{\varepsilon }{1+\varepsilon }}$$

where ε is the tensile strain of high-performance fabric.

The above equations demonstrate that a high modulus and a lower density facilitate the more efficient proliferation of stresses through the yarns. Hence, an increased yarn count enhances energy absorption and improves energy dissipation when subjected to ballistic impact loads.

## 3. Wave Transmission Through the Fabric Under an Impact Load

The fabric’s ballistic behavior and wave propagation are similar to a single yarn. Woven fabrics under impact loading experience deformation, characterized by longitudinal strain within the plane and transverse strain outside the plane. Initially, the fabric experiences a minor strain in the impact zone, which further propagates to the adjacent yarns along both the yarn and transverse axes [2]. The waves propagating along the yarn axes are termed longitudinal strain and are uniformly distributed at low-impact velocities. However, at higher impact velocities, this may not be effective. Earlier studies indicate that strain waves vary with increasing impact velocity. On the other hand, out-of-plane waves pushed away the fabric in the direction of the impacted area to help in maximum energy dissipation. The relationship between impact velocity and fabric strain is described by Equations (3) and (4) [39].

(3) $$\varepsilon =\frac{V}{c}$$

(4) $$c=\sqrt{\frac{1}{\rho }}$$

where *V* is the speed of projectile in m/s, *c* is the impact velocity in m/s, ρ is the mass density of the material, and *ε* is the strain.

### Impact Energy

The energy absorption characteristics of high-performance fabrics depend on the amount of energy they absorb during impact. The energy absorption during the impact can be calculated by Equation (5) [40].

(5) $$U=\frac{1}{2}m\left({v_{1}}^{2}-{v_{2}}^{2}\right)$$

where *U* is the energy dissipation in Joules, m is the projectile’s mass in kg, *v*_1_ is the initial velocity, and *v*_2_ is the final velocity in m/s.

This equation specifies the ballistic limit of the soft body armor. When the projectile fully penetrates the material, its exit velocity is recorded with a high-speed camera or, occasionally, a chronograph. Furthermore, the energy transferred from the projectile and absorbed by the fabric panels occurs through various mechanisms, such as yarn pull-out, fiber fracture, fiber type, areal density, weave pattern, surface finish, number of fabric layers, and stacking sequence [41], which are elaborated in detail in the following sections.

## 4. Key Variables in Impact Energy Absorption

The energy absorption phenomenon in fibrous materials is a complex process that involves numerous parameters. The selection of high-performance fabrics (Kevlar and UHMWPE) is crucial for armor design, as they absorb the maximum impact energy. Fiber properties are greatly affected by areal density, weave design, crimp, yarn twist, the number of threads/length, and assembly parameters like ply orientation, the number of layers, and ply combinations [42]. Additionally, different fiber structures, such as unidirectional (UD), two-dimensional (2D), and three-dimensional (3D), are vital for maximizing energy absorption [42,43]. UD fabrics consist of filaments oriented along the length of the fabric, without interlacing, resulting in a crimp-free structure. In contrast, 2D fabrics have filaments interlaced in two directions, while in 3D fabrics, yarns are arranged in three directions [44]. Apart from fabric properties, projectile variables, namely, mass, velocity, and geometrical characteristics, also influence the fabric’s energy-absorption performance. In Section 4.1, an attempt has been made to elaborate on all the parameters that directly or indirectly affect the fabric when subjected to impact loads.

### 4.1. Fiber Properties

Fiber properties significantly influence the impact performance of the fabrics. Various physicochemical properties significantly impact the energy absorption and stability of fabrics under high-velocity loads [45]. Some of the high-performance fabrics used to develop protective systems are described in the following section.

#### 4.1.1. Aramid Fibers

**Figure 7:**
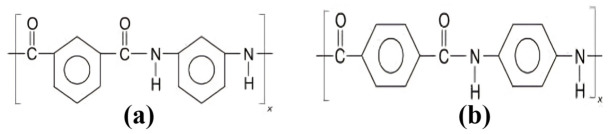
Chemical structure of aramid fiber: (**a**) meta-aramid and (**b**) para-aramid (adapted from [12], Scientific review, 2021).

Aramid fibers possess superior mechanical properties compared to steel and glass fibers per unit weight [46]. Moreover, these fibers possess inherent heat- and flame-resistant properties, making them suitable for elevated temperature conditions up to 500 °C [47]. Aramid fibers are composed of aromatic polyamide with 85% amide bonds (-CO-NH-) attached between two aromatic rings, as depicted in Figure 7 [12]. Based on chemical linkage, there are primarily two aramids, meta-aramid (Nomex) and para-aramid (Kevlar and Twaron) [48]. Para-aramid outperforms meta-aramid in terms of mechanical properties due to structural differences.

The first aramid fiber was developed in the 1960s by DuPont and was initially used to produce fire-resistant clothing. Later, the brand name changed to Kevlar, which contains *p*-distributed benzene rings with superior mechanical properties. The chemical composition of Kevlar and Twaron fiber is poly (p-phenylene terephthalamide), which is synthesized from two monomers: 1,4-phenylenediamine and terephthaloyl dichloride, using complex solvents. The aramid fabric is made through polymerization, filament yarn spinning, and staple fibers. There are several types of commercial aramid fibers, including Kevlar 29, Kevlar 49, and Kevlar 149. Kevlar 49 is the most popular fiber for its high modulus, whereas Kevlar 29 is known for its high toughness, damage resistance, and impact resistance. Kevlar 149 offers superior mechanical properties compared to Kevlar 29 and Kevlar 49, as summarized in Table 1.

#### 4.1.2. Ultra-High-Molecular-Weight Polyethylene

**Figure 8:**
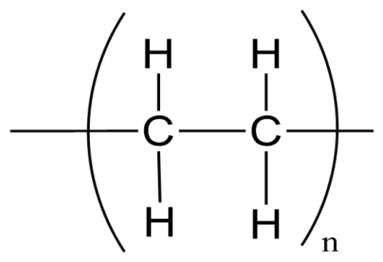
Chemical structure of UHMWPE (adapted from [13], Zastita Materijala, 2024).

Apart from aramid fibers, UHMWPE (Spectra and Dyneema) was also evaluated for ballistic protection systems due to its exceptional mechanical performance and reduced weight [51,52]. UHMWPE is made of a long polyethylene chain with a molecular weight of 28 and was first made available in the 1980s by Honeywell Advanced Fibers and Composites, USA, and DMS High-Performance Fibers, The Netherlands [53]. The molecular structure is illustrated in Figure 8 [13].

Compression molding, ram extrusion, and gel-spinning stand out among the various methods for UHMWPE production. However, the gel-spinning process is usually preferred for body armor production, in which a gel material forms when dissolved ethylene is drawn through several tiny openings [52]. Fibers produced by gel spinning offer enhanced toughness and more resistance to chemicals and abrasion. The strength-to-weight ratio of UHMWPE was found to be 40% greater than that of para-aramid fibers for the same weight ratio, meaning it is a feasible fiber for developing soft body armor. However, UHMWPE exhibits a 150 °C melting point, lower than the para-aramid fibers, and showcases weak softening and easy creep under high loading [2]. The properties of UHMWPE are presented in Table 2.

#### 4.1.3. Other Fabrics

This section highlights the fabrics that are not greatly suitable for impact application but which have been explored for impact applications. These fabrics are explained in the subsections.

##### Glass Fiber

In 1893, the mass production of glass fibers was initiated by Edward Drummond Libbey as a dress crafted from silk and glass fiber; the first patent of glass wool was proposed by Russell Games Slayter in 1938. The produced glass fiber exhibited good electrical conductivity; hence, the name was called electric glass or E glass. Since 1939, glass fibers have been utilized as insulators, structural parts, and aircraft [14,21]. The manufacturing of E-glass includes silica, aluminum oxide (Al_2_O_3_), boric acid, and limestone, which are set in the furnace at 1600 °C [22]. As molten glass leaves the furnace into the forehearth channel, fibers form. Further, the fiber diameter can be adjusted when fibers flow through thousands of tips (nozzles) at that time. The desired fibers are quenched by cooled air or water spraying, and after that, aqueous size is applied to the fibers to protect the thin filaments from abrasion. Different types of glass fibers, such as A, C, D, E, AR, R, S, and S-2, are known for their unique properties and for having different chemical positions [23]. Among all types, E and S glass have been investigated for composite fabrication due to their excellent tensile strength, as depicted in Table 3 [54]. Glass fibers and their composites are being utilized in electronics, aviation, medical, automobile, aesthetic, and low-velocity response applications [24].

It is evident from Table 1 that the density of glass fiber ranges between 2.48 and 2.58, which is approximately 42% higher than aramid and 61% higher than UHMWPE fibers. However, the fracture strain (%) of glass fiber is higher compared to the aramid and UHMWPE fiber, meaning these fibers are not suitable for ballistic body armor due to poor energy absorption and toughness [25].

##### Carbon Fibers

Carbon fibers offer the highest specific strength and modulus among all fibers. The carbon fibers do not exhibit stress rupture failures like glass and organic polymer and withstand their properties at elevated temperatures [26]. Carbon fiber contains at least 90% of carbon obtained through a controlled pyrolysis process using suitable fibers. Carbon fibers can be produced by converting the polyacrylonitrile (PAN) precursor into high-performance carbon fibers. The process involves three stages: (a) Oxidative stabilization in which PAN is stretched first and then oxidized in a temperature span of 200–300 °C. In this stage, PAN transforms into a non-plastic cyclic or ladder structure. (b) Carbonization: At this point, fibers undergo carbonization at 1000 °C in an inert atmosphere for a few hours, eliminating any undesired components from the carbon fibers. In the last stage, (c) Graphitization: fibers are set at the temperature range of 1500 and 3000 °C, improving the crystallites’ orientation [19]. Eventually, graphene sheets are formed with 93–95% carbon. The physical and mechanical properties of the based carbon fibers are summarized in Table 4.

Further, carbon fibers can be produced from pitches derived from petroleum-based residues. Pitches are thermoplastic materials extruded through a melt-spinning process to precursor fibers [28]. The production routes to develop carbon fiber using these fibers are similar to the PAN route; however, the stabilization step lies in the temperature span of 250 °C and 300 °C, whereas carbonization and graphitization occur between 1000 °C and 2500 °C. Carbon fibers can be produced from rayon fibers obtained by dissolving and spinning cellulosic materials (wood pulp or cotton). The stabilization temperature is below 400 °C; subsequent carbonization occurs at 1500 °C, and eventually graphitization at about 2500 °C. The carbon fiber yield from rayon fiber contains a carbon percentage of 10 to 30 wt.% [3].

These fibers are valued for their ability to withstand high temperatures, provide effective damping, conduct electricity and heat efficiently, and resist chemical degradation [4]. Previous studies reported that carbon fibers have an exceptional stiffness and modulus, and they are low-density, making them ideal for developing protective gears (helmet, automobile parts, and military applications) but not for typical body armor due to low toughness and high brittle behavior, which leads to catastrophic failure in carbon fiber composites against a high impact velocity [5,15].

##### Ceramic Fibers

Ceramic fibers in long and short forms have been commercially available since the 1970s. They are valued in engineering applications thanks to their high structural resilience, mechanical strength, and creep resistance properties at elevated temperatures. The most known oxide ceramic fabrics, such as aluminum oxide (Al_2_O_3_), silica (SiO_2_), and zirconia (ZrO_2_), are suitable for superior insulating properties compared to non-oxide ceramic fibers [16]. Oxide ceramic fiber offers low thermal conductivities, excellent thermal shock resistance, insulation, and acoustic properties. The manufacturing process, namely, slurry spinning, sol–gel spinning, and single crystal growth, are used to manufacture the oxide ceramic fibers. The fabrication of non-oxide ceramic fibers, including SiC, silicon carbon-nitride (SiCN), boron carbide (B_4_C), and boron nitride, is complex because of their high melting points and resistance to densification. Table 5 highlights the properties of some ceramic fabrics. Most non-oxide ceramic fibers are produced using the chemical vapor deposition (CVD) method [17].

From the point of view of industrial implementation, ceramic fiber composites are employed in aircraft components, gas turbine parts, missiles, heat exchangers, rocket nozzles, gaskets, and insulators [18]. Recently, Jiang et al. [55] reported that B4C plate armor outperformed SiC armor plate, improving overall ballistic performance.

##### Zylon

**Figure 9:**
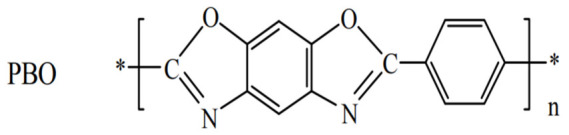
Chemical structure of PBO (adapted from [29], MDPI, 2008).

Poly(p-phenylene-2,6-benzobisoxazole), commonly known as Zylon, is a liquid crystal thermoset polymer. Zylon was invented in 1980 by Toyobo Japan [56,57]. Zylon has been identified as a potential candidate for replacing Kevlar due to its superior tensile strength and thermal durability [58]. The first study of the synthesis of PBO was proposed by Wolfe et al. [20] using polycondensate 4,6-diaminoresorcinol hydrochloride (DAR·HCl) and terephthalic acid (TPA) derivates in the presence of poly(phosphoric acid) (PPA). The chemical structure of PBO is illustrated in Figure 9 [29].

The physical and mechanical properties of poly(p-phenylene-2,6-benzobisoxazole) are summarized in Table 6.

**Figure 10:**
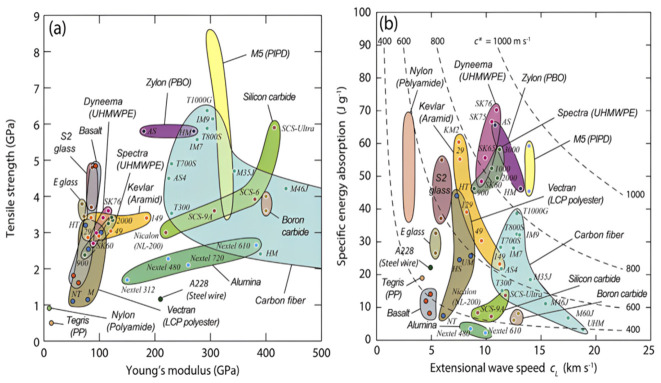
Material property charts: (**a**) tensile strength against Young’s modulus and (**b**) specific energy absorption vs. velocity of propagation of the longitudinal wave (equation 2.1) of high-performance fibers. Contours of the Cunniff velocity, c*, are also plotted in (**b**) (reprinted with permission from [59], Elsevier, 2014).

To maximize body armor protection, a combination of high tensile strength, elastic modulus, superior energy absorption, and low density is essential. A comparative analysis of advanced materials used in protective gear and structural materials is shown in Figure 10 [59]. Figure 10a shows the relationship between Young’s modulus and tensile strength, while Figure 10b represents the plot between specific energy absorption and extensional wave speed. The stiffness and high strength of M5, Zylon, and carbon fibers (T1000, IM7) stand out among other materials for the development of high-performance applications. Apart from these fabrics, aramid fibers (Kevlar), UHMWPE (Dyneema, Spectra), and basalt fibers offer high tensile strength and moderate stiffness, making them ideal for flexible and durable applications [60]. Ceramics such as silicon and boron carbide exhibit excellent stiffness but low to moderate tensile strength. Moreover, specific energy absorption and the extensional wave speed are decisive for impact-resistance applications. UHMWPE, Zylon, and Kevlar exhibit exceptional energy-absorption capabilities [61]. High-performance materials exhibit higher tensile strength and specific energy absorption than traditional fibers in both plots. Together, the plots highlight the trade-offs and assessment criteria for armor materials.

### 4.2. Yarn Parameters

Yarn is a long strand of material made from multiple synthetic or natural fibers. Yarn characteristics, such as twist and yarn friction, influence the fabric’s impact properties. The section provides a detailed explanation of these parameters.

#### 4.2.1. Yarn Twist

The yarn becomes stronger when twisted, due to the firm binding of its fibers. Twisting generates radial forces that draw the yarns together more cohesively. It is well established that twisting increases the strength of staple fibers positively, but after reaching a specific value, the strength declines due to the obliquity effect. In multifilament high-performance fibers, a small degree of twist is typically applied to improve handling and prevent de-filamentation [62]. Rao and Faris [63] found that the strength of Kevlar 49 increases with an increasing twist angle, whereas Spectra fiber shows only a minimal increase in strength. However, the rise in strength across all yarns (Kevlar 29, 49, 129, Spectra, and Technora) showed a maximum at a twist angle of 7°. In another experimental work, Pan et al. [64] observed a decrease in the breaking load of Kevlar 49, from 8.16 kg to 7.41 kg, when its twist angle was increased from 0° to 10°. Hence, a twist angle of less than 10° proved most effective for fiber performance and elevated yarn strength.

Later on, the impact of yarn twist on the mechanical performance of s-glass yarn and their composites was investigated by Dalfi et al. [31]. They reported that twisting helps in improving the mechanical performance of S-glass yarn composites. However, the tensile properties improved up to 30 twists meter^−1^, while it declined at 40 twists meter^−1^. The same study was performed by Jiang et al. [32] with nylon/aramid-wrapped yarns and their fabrics. The outcome of this study revealed that twisting yarns significantly enhanced the mechanical properties especially; at a core yarn twist level of 80 turns per meter, the tensile strength increases by 20.6% compared to untwisted core yarns.

#### 4.2.2. Frictional Resistance

**Figure 11:**
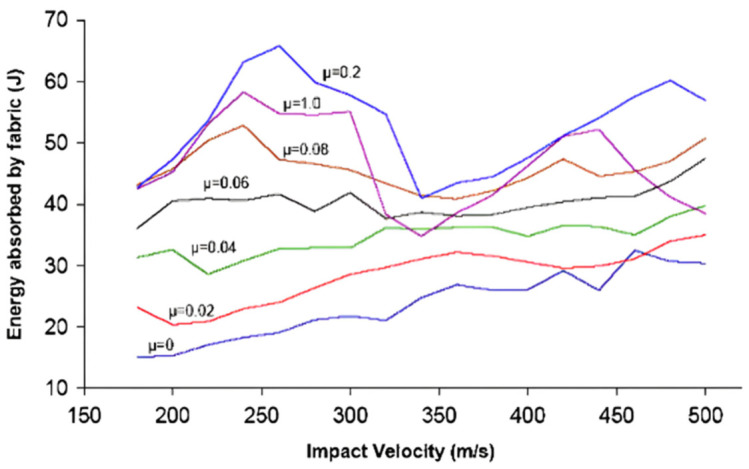
Impact energy absorption response of fabric with different coefficients of friction (reprinted from [38], RCS Advances, 2019).

The ballistic response of fibrous body armor is governed mainly by yarn–yarn friction. Most multifilament fabrics are smooth, shiny, and greasy, resulting in a low coefficient of friction. The fabric with a low frictional coefficient exhibits low shear resistance upon impact with high-speed projectiles, a measure of the yarn–yarn friction of the fabric. The inter-yarn frictional resistance can be significantly improved by removing the size, using resins or adhesives, and incorporating different nanoparticles. Improving yarn-to-yarn frictional resistance involves modifying the yarn surface to enhance energy absorption and integrity. The impact of friction on ballistic energy absorption was analyzed by Duan et al. [65] using FEA. During their numerical work, they discovered that for a zero value of the coefficient of friction (µ), the fabric structure is significantly deformed at the impacted region and near the principal yarns. Meanwhile, for µ = 0.5, the fabric deformation is restricted to the impacted region only. In continuation, Zeng et al. [66] also investigated the inter-yarn frictional resistance of fibrous armor using computational modeling. They carried out their investigation over a range of friction coefficients (µ = 0 to 1). It was reported that low-friction µ = 0.02 fabric is easily penetrated by the projectile. In contrast, at high friction (µ = 1), the fabric’s mobility is restricted. It implies that high frictional resistance helps in maintaining fabric integrity by hindering the yarn movement within the fabric when impacted. The impact energy absorption by the fabric with respect to different impact velocities and coefficients of frictional resistance is depicted in Figure 11 [38]. The graph indicates that high frictional resistance leads to improved energy absorption.

**Figure 12:**
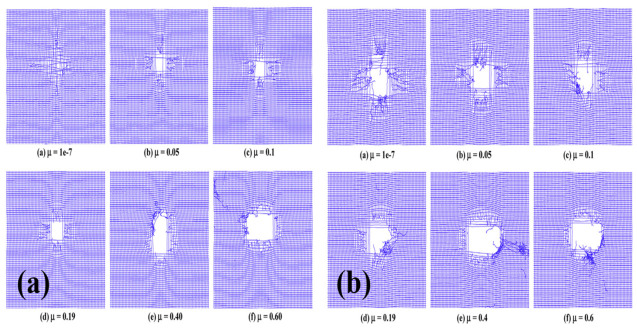
Influence of coefficient of friction of fabric on failure mechanism for round (**a**) and flat nose (**b**) projectiles: (**a**) µ = 1 × 10^−7^, (**b**) µ = 0.05, (**c**) µ = 0.1, (**d**) µ = 0.19, (**e**) µ = 0.4, and (**f**) µ = 0.6 (reprinted with permission from [67], Elsevier, 2015).

Further, the role of frictional resistance against different impactors was investigated by Das et al. [67] on woven fabric for low-velocity impact using flat and round-nose projectiles. They observed that the fabric penetration behavior changes with the µ for the round nose projectile. In contrast, the fabric penetration was almost the same for the flat projectile, irrespective of the change in µ. Figure 12 [67] shows the fabric deformation by round and flat projectiles at different coefficients of friction.

#### 4.2.3. Yarn Evenness

Yarn evenness refers to the variation in the properties, such as twist, strength, thickness, and fineness along the length. Such variations in the fibers are inevitable because they came into existence from the resulting arrangement [68]. In one study, the effect of sizing on yarn properties was investigated by Krstovic et al. [69] using two sizing agents (FibrosintC75 and Inex773C). The outcome revealed that sizing helps in improving yarn evenness by lowering irregularity, uniformity, and smooth yarn structure per unit length. Recently, Bekinew Kitaw Dejene and Million Ayele [36] addressed that yarn evenness leads to less irregularity, structural variations, instability, and inconsistent performance.

### 4.3. Fabric Parameters

Fabric parameters, such as areal density, weaving architecture, number of plies, and ply sequence, influence the performance and structural integrity of a fabric under ballistic loads. The subsections elaborate on how different weave patterns respond to the impact energy absorption. Additionally, the section explains the significance of the number of fabrics and their orientation in relation to impact loads.

#### 4.3.1. Weave Architecture

**Figure 13:**
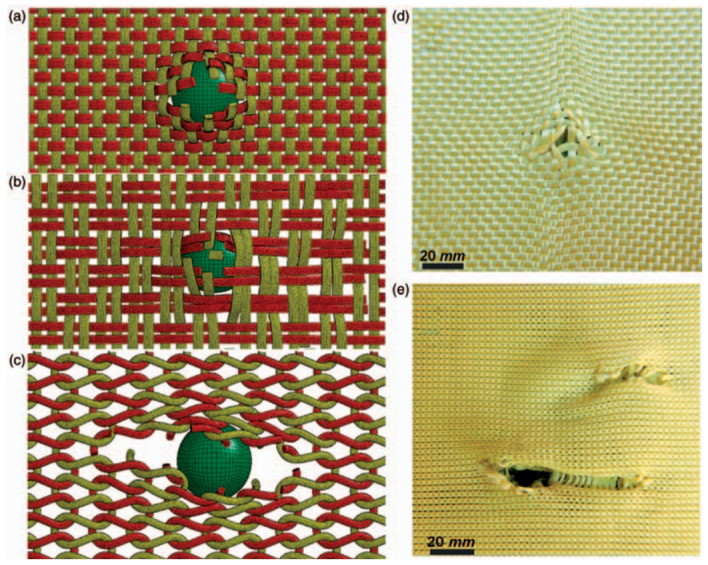
Bottom view of ruptured fabric: (**a**) plain woven, (**b**) basket woven, (**c**) knitted fabric, along with experimental data for (**d**) woven and (**e**) knitted fabrics (reprinted with permission from [2], Elsevier, 2019).

Weave architecture significantly affects both the mechanical performance and energy-absorption capabilities of fabrics. Various weaving patterns, including plain, basket, twill, and satin, are available and have been used for ballistic applications [70]. One of the earlier studies by Cunniff et al. [71] on the ballistic response of Kevlar 29 and Spectra with varying parameters confirmed that the energy-absorption capacity of nylon fabric is far inferior to that of Kevlar and Spectra fabrics due to the low strength-to-weight ratio. However, the study confirmed that the impact energy absorption for both Kevlar and Spectra is influenced by weave pattern and fabric density. Recently, Tran et al. [70] explored the FEA modeling of three common weave patterns: plain, basket, and knitted fabric. They found that knitted fabric exhibited the worst performance in ballistic applications among the selected fabrics, as the crack propagation in this fabric was observed earlier due to the significant transverse shear loading. On the other hand, 2 × 2 basket fabric also exhibited similar ballistic resistance as knitted fabric; only plain weave fabric outperformed in energy absorption during impact loads [70]. The failure pattern in plain woven, basket woven, and knitted fabrics is shown in Figure 13 [2]. It is evident from Figure 13 that fiber rupture is very prominent in knitted fabric compared to the rest of the fabrics.

In continuation, Yang et al. [72] also performed numerical analysis for four different woven fabrics: plain, 2/2 basket, 2/2 twill, and 4-harness satin weaves. The study validated that the plain weave fabric exhibited a superior performance under ballistic loads due to its strong interlacing and resistance to abrasion. The strong interlacing of plain weave fabric resists yarn slippage. It uniformly distributes impact forces, which is superior to other fabrics due to their poor interlacing and localized deformation during impact loads. Further, the influence of weave architecture on 3D orthogonal interlock woven fabric was examined by Wu et al. [73]. They found that the 3D weave pattern has a limited effect on ballistic performance due to high structural integrity and high strain-rate dynamics.

#### 4.3.2. Number of Fabric Layers

**Figure 14:**
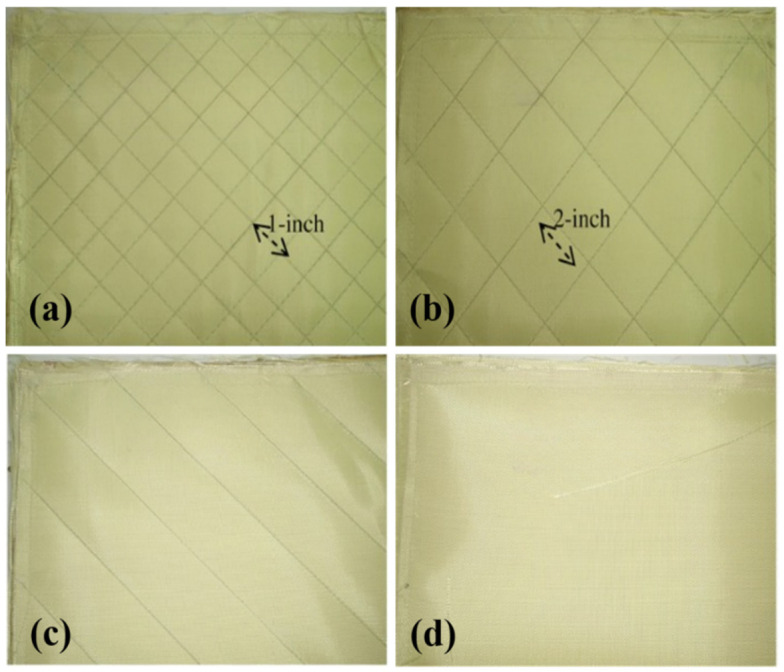
Different stitched fabric systems: (**a**) 1-inch diamond, (**b**) 2-inch diamond, (**c**) diagonal, and (**d**) perimeter (reprinted with permission from [76], Elsevier, 2008).

Previous research has addressed the complex mechanisms behind projectiles’ penetration and perforation of targets. As the single-layer high-performance fabric fails to meet the requirements of restricting fabric penetration, multi-layer fabrics have become necessary in designing armor panels. The study by Karahan et al. [41] focused on the ballistic performance of woven and unidirectional fabric panels with varying fabric layers. They found that the areal specific energy absorption declined with an increase in the layer count. K-Flex unidirectional fabric panels demonstrated a 12.5–16.5% higher impact energy absorption than Twaron fabrics at the same weight per unit area. Likewise, an experimental study was performed by Nguyen et al. [74] to investigate the deformation and failure mechanisms of UHMWPE impacted by fragment-simulating projectiles of varying thicknesses (9–100 mm). Thin panels (thickness 9 mm) were found to be more vulnerable to the impact, resulting in large deflection and bulging, with an increase in the thickness of panels. This demonstrated two-stage penetration, with shear plugging at the preliminary stage, succeeded by bulging of the panel in the second stage. The study aimed to examine the impact energy absorption performance of body armor constructed from 3D warp interlock and 2D plain woven aramid fabrics [75]. From the test, it was evident that the ballistic performance of the 3D warp interlock fabric was not on par with the 2D plain fabric. Both 40 and 30 layers of 2D plain woven fabric demonstrated superior energy absorption capabilities compared to 3D warp interlock fabrics and their counterparts. Additionally, an investigation was performed into how fabric stitching modifies ballistic performance. The ballistic behavior of natural fiber-coated fabrics under the effect of stitching was assessed by Ahmad et al. [76]. They employed 32 and 28 layers of neat, unstitched Kevlar 29 fabrics, as well as 26- and 24-layer natural rubber-coated stitched (diamond) and unstitched fabrics for analysis. The different stitched fabric systems are shown in Figure 14 [76].

The experimental results showed that different stitching patterns (2-inch field diamond, diagonal, and perimeter) provide better ballistic resistance than the 1-inch field diamond stitch and the unstitched fabric system. The 1-inch field diamond stitch fabric features a much denser stitching pattern than the 2-inch stitching and thus acts as a stress concentration zone during impact. On the other hand, unstitched fabric is unable to offer sufficient resistance against impact. In this direction, Bilisik et al. [77] conducted an experimental investigation to analyze the effect of stitched and unstitched aramid fabric systems on yarn pull-out tests. They reported that stitching improves the pull-out forces compared to the unstitched fabrics. Moreover, it was also found that stitching makes the structure stiff and reduces fabric deformation. Later, Zhao et al. [78] reported the superior ballistic performance of stitched plain fabric. In previous studies, the importance of several fabric layers and stitching was revealed when subjected to ballistic loads.

#### 4.3.3. Ply Arrangement/Orientation of Fabric Layers

**Figure 15:**
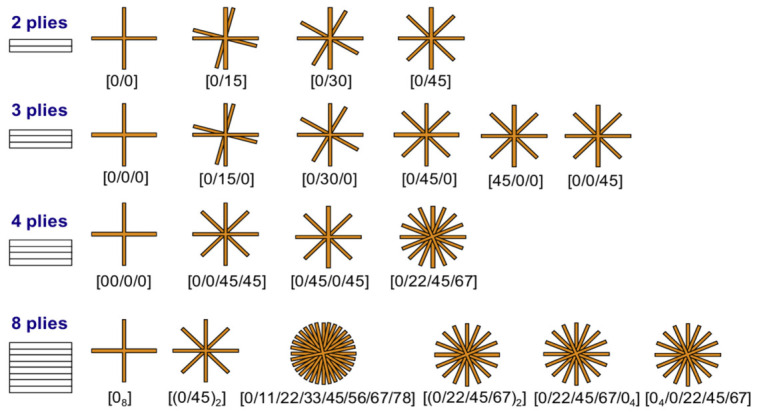
Different fabric sequences are used in the FEA simulation (reprinted with permission from [80], Elsevier, 2015).

A range of fabrication methods can increase the ballistic efficiency of fabric panels used in armor system, including the use of stacking sequences and combinations of materials [79]. The arrangement of plies in fabric panels is critical to their energy absorption performance under ballistic impact. Cunniff et al. [71] demonstrated the influence of stacking sequences using a double-ply system. They used two systems; the first was composed of 1000 denier Kevlar 29 fabrics, followed by 375 denier Spectra fabrics, and had a ballistic limit of 269 m/s. On the other hand, an inverted ply sequence with a ballistic limit of 114 m/s was applied. The experimental results for energy absorption show no effect when the same type of material is used for the first and second plies. However, a notable difference in energy absorption was observed in the ballistic test across varying ply orders. The results of this study were found to be promising for double-ply panels, and further investigation was conducted on multi-ply panels to examine the role of the stacking sequence. In this quest, a numerical study on the ply orientation of multi-ply panels for ballistic resistance was conducted by Wang et al. [80]. They used 2, 3, 4, and 8 plies in different orientations using finite element modeling, displayed in Figure 15 [80]. The role of fiber layup on the energy-absorbing capacity of multi-ply panels was found to be significant. Moreover, it was noticed that a correct position of angle plies is needed to elevate the energy absorption potential of panels. An 8-ply with [0/22.5/45/67.5]_2_ order was found to be suitable for the best impact performance.

In this continuation, Min et al. [81] investigated the impact of angled-ply orientation on the ballistic performance of multi-ply UHMWPE panels. They used 3-ply aligned-laid [0/0/0] and angled-laid [0/30/60] fabric systems for their investigation. The angled-laid panels were found to be more energy-absorbent compared to aligned-laid panels owing to the better stress distribution in multiple directions with minimal yarn slippage. In the latest research, Peinado et al. [82] investigated the UHMWPE fabric to assess how stacking sequence variations influence performance. They used 17 stacking sequences with three different UHMWPE fabrics in modulus and areal densities, namely PE1, PE2, and PE3. Thus, it can be concluded that the placement of fabric can influence the ballistic limit of the panel during high-velocity impact. Therefore, various stacking sequences have been investigated by researchers to optimize impact performance and develop a feasible armor material.

#### 4.3.4. Hybridization of Fabric

The ideal fiber materials for ballistic use are those with high strength and modulus and low density. However, various limitations of these fiber materials motivate researchers to find alternative approaches to develop a more feasible protection system [83]. In the previous section, the importance of fiber arrangement and orientation has been addressed. Like ply orientation, fiber arrangement is also crucial in influencing ballistic performance. Therefore, nowadays, hybrid panels are often chosen for ballistic shields, attributed to their lightweight design and excellent impact performance. Hybridization is the process of combining two or more different types of fabrics to achieve tailored properties that cannot be obtained from single fabric alone. Researchers globally have evaluated the effectiveness of hybrid panels compared to single-material panels and have confirmed that hybridization enhances the ballistic performance against various projectiles [84]. The impact resistance of hybrid panels was showcased by Pandya et al. [85]. Four hybrid laminates consisting of 8H satin carbon fibers and plain weave E-glass reinforced with epoxy resin were examined. Hybrid composites showed a higher ballistic limit velocity than carbon composites at equal thickness.

Additionally, applying E-glass as the exterior layer while keeping carbon as the interior layer raises the ballistic limit velocity. Furthermore, the impact resistance of the hybrid panel composed of plain and UD Dyneema fabric was investigated through numerical and experimental studies by Chen et al. [86]. Type A panels, where woven fabrics were positioned in front of UD fibers, and Type B panels, featuring reversed arrangements, were analyzed. A configuration consisting of 6 woven fabric layers and 30 unidirectional layers produced the minimum back-face signature. In comparison, 40 layers of UD fabric and 12 layers of woven fabric, combined with 20 layers of UD fabric, resulted in similar penetration depths of approximately 8.5 mm. The 24 layers of woven fabric showcased the worst performance during ballistic analysis. The outcome of this study shows how hybridization helps to better the impact performance of panels. Researchers have assessed the ballistic impact response of hybrid natural/synthetic fibers, yielding outstanding results from hybridization with synthetic fibers [87]. Yahaya et al. [88] analyzed the ballistic limit velocity (V50) and energy absorption by kenaf/aramid hybrid composites. They found hybridization results in lower specific energy absorption than non-hybrid Kevlar composites. Woven bamboo/E-glass polyester hybrid composites were also examined for their ballistic response [89]. The study found that 4/18 layers of woven bamboo/E-glass withstand a bullet velocity of up to 482 m/s and hence make it suitable for the NIJ IIIA standards, whereas 9/4/9 layers of E-glass/woven bamboo/E-glass are only ideal for NIJ level II protection at 414 m/s.

#### 4.3.5. Directional Yarn Crimp

Crimp is the inherent undulation in yarn resulting from the weaving process. Yarn crimp is unwanted yet inevitable in woven fabric for ballistic applications. Yarn crimp has been identified to have a crucial impact on wave propagation during ballistic loading [71]. The fabric with high crimp exhibits less resistance to projectiles as the yarn stretches effortlessly. Moreover, a high crimp allows for more deflection along the lateral axis and even increases the risk of blunt trauma. Interestingly, in some studies, plain weave fabric with higher crimp outperformed other weaves (twill, satin, and matt). This highlights the optimal relationship between contact points and crimp intensity. Ballistic high-performance fabrics typically exhibit different warp and weft patterns due to their weaving process, and the weft yarn has a lower crimp than the warp yarn. It is believed that weft yarn is more likely to break than warp yarn when subjected to ballistic loading. Chitrangad et al. [90] suggested using warps with immense failure strain so that both yarns break simultaneously during impact. Unlike this, Sadegh and Cavallaro [91] found that an optimum crimp imbalance in woven fabrics could affect the energy absorption for a fully perforating impact. Their research showed that a crimp-balanced structure was more effective in reflecting outward wave energy at the impact site; however, the wave transmission from the impact area was delayed with a higher crimp in fabric. Additionally, this delay makes stress wave distribution less effective at the yarn intersection points. Nevertheless, obtaining a flawlessly balanced structure is practically unfeasible; a certain amount of crimp exists in the fabric. This was numerically investigated by Tan et al. [92]. A closer match to the actual results was achieved when zigzag elements rather than straight lines represented the crimp.

### 4.4. Fabric Architecture

The fabric architecture serves an important function in protective systems, absorbing and dissipating energy during impact loadings. High-performance fabrics selected for protective gear should have a high strength and modulus and excellent anti-degradation traits. These traits are crucial for reducing projectile impact velocity, deforming the projectile’s shape, and eventually restricting the projectile to the structure. The market currently offers diverse types and configurations of ballistic fabrics, which include UD, 2D, and 3D [93]. Every fabric structure, elaborated upon in the subsections, demonstrates a unique suitability for advanced protection.

#### 4.4.1. Unidirectional Structure

In UD fabrics, fibers are arranged exclusively along the longitudinal axis and held together by another filament or by some laminating films to keep the fibers intact [86,94,95]. UD fabric retains high mechanical strength along the axis of its inlaid yarns, and therefore, UD fabric composites are used in 0° and 90° orientations for ballistic applications. As UD fabrics are free from crimp, the speed of the shock wave is expected to be higher and smoother during impact [96].

#### 4.4.2. Two-Dimensional Structure

Two-dimensional fabric structures are composed of two sets of yarns, generally called warp and weft, interlaced in a woven structure, looped in a knitted structure, or sometimes braided or stitched in a repeating pattern [97]. The detailed technical specifications are given in the subsections.

##### Two-Dimensional Woven Fabric

**Figure 16:**
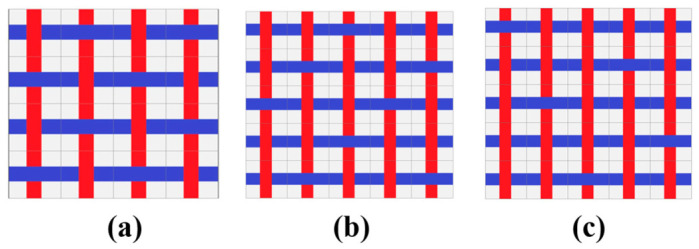
Basic 2D woven fabric configurations: (**a**) plain, (**b**) twill, (**c**) satin (reprinted from [39], MDPI, 2022).

Two-dimensional woven fabric finds extensive use in ballistic-resistant designs to counter threats such as ballistic, stabbing, and spike attacks. The basic configurations of 2D woven fabrics are depicted in Figure 16 [39]. The weft and wrap interlace over and under each other in plain fabric, resulting in a stable, symmetric fabric with a significant crimp. Additionally, basket weave is a plain fabric featuring multiple interchangeable warps and weft yarns. However, the amount of crimp reduces in the basket-weave fabric. In twill fabric, warp yarns pass alternately over and under weft yarns, resulting in a smooth fabric surface with low crimp. The high in-plane properties of 2D plain, basket, and twill fibers make them ideal for designing soft body armor. In satin fabric, warp yarns alternately cross over under weft yarns, resulting in several interactions. Owing to their low crimp, satin fabrics are effective in constructing rigid body armor [98]. The leno weave is another construction often used with other weave patterns. The adjacent warp yarn is twisted around successive weft yarns in a leno weave fabric. These fabrics are beneficial for constructing mosquito netting and insect prevention fabrics [99].

##### Two-Dimensional Knitted Fabric

**Figure 17:**
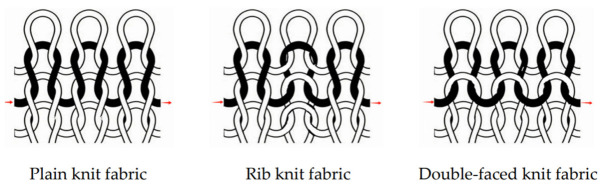
Different 2D knitted fabric configurations (reprinted from [40], MDPI, 2025).

Knitting refers to the process of producing fabric by interlocking loops of yarn with needles. In a knit structure, rows of fabric are called courses, whereas columns are called wales. Depending on the direction in which loops are knitted, there are two primary types of knitted fabric: warp and weft-knitted fabrics [100,101,102]. Figure 17 [40] shows the basic schematic of both structures. Knitted fabrics are superior in energy absorption, low resistance to deformation, and fracture toughness because of their looped and flexible structures. However, the strength and stiffness of knitted fabrics are usually inferior to woven, braided, and non-crimped fabric materials. Warp and weft knitted yarns can be reinforced bidirectionally by incorporating inlay yarns, which enhance the structural integrity and ultimately increase the mechanical traits of the fabric and its composites [103,104].

##### Two-Dimensional Nonwoven Fabrics

Nonwoven fabrics are sets of irregularly arranged fibers or chopped yarns used for innovative, cost-effective solutions to many engineering problems. The fibers in nonwoven fabric can bond together through chemical, mechanical, and thermal bonding. Mechanical bonding is the primary method for stiffening fibers and uses hydro-entangling, stitch-bonding, and needle-punching [105].

#### 4.4.3. Three-Dimensional Fabric Structure

**Figure 18:**
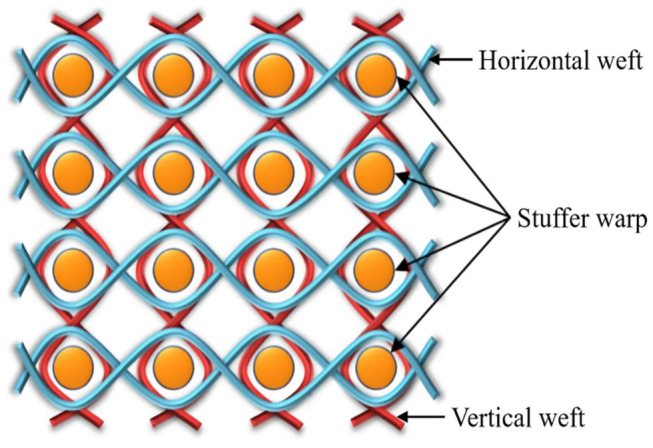
Schematics of actual 3D woven fabric (reprinted from [106], Oxford Open Materials Science, 2023).

Recently, 3D fabrics have been used in ballistic applications. Three sets of yarns weave in a three-dimensional fabric structure in three different directions, depicted in Figure 18 [106]. A series of yarns positioned in the warp direction is also called stuffer warp, the other is in the weft, and the third set of yarn runs throughout the thickness direction (vertical weft or Z direction) [106].

**Figure 19:**
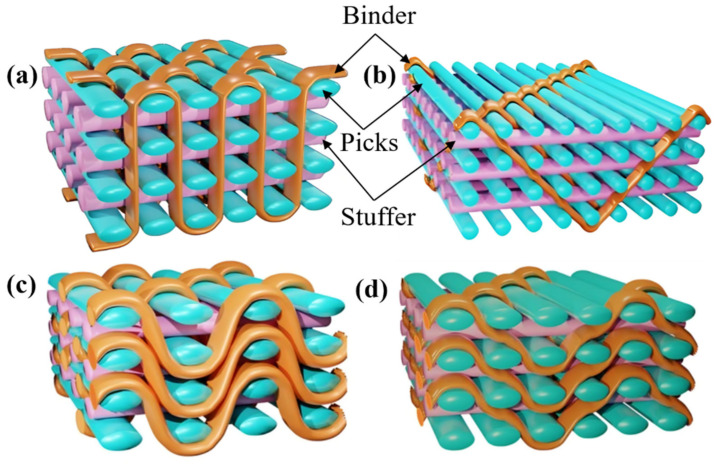
Schematics of non-interlaced 3D fabric structures: (**a**) Orthogonal 3D angle-interlock through thickness, (**b**) Layer-to-layer angle-interlock, (**c**) 3D angle interlock layer to layer, and (**d**) 3D angle interlock through thickness (reprinted from [106], Oxford Open Materials Science, 2023).

When a 3D fabric is crafted using a traditional 2D loom, a noninterlaced 3D fabric structure (‘noobed’ configuration) is developed. Stuffer and binder yarns are arranged longitudinally but extracted from separate beams [107]. Binder yarns keep the entire fabric structure intact in the thickness direction, known as through-thickness interlocking. When binder yarns are aligned at an angle, it is referred to as an angle interlock structure [108]. If there are layers between the weft, it is called a layer-by-layer interlocked structure. Hence, there are four primary types of 3D woven fabrics, illustrated in Figure 19 [106]. Over time, the terminology for different 3D fabric structures was revised, as listed in Table 7, to prevent miscommunications.

Owing to their high mechanical properties and energy-absorption capabilities, 2D plain fabrics are frequently chosen for the design of soft body armor. However, the concentration of localized stress at intersection points and stress distribution during ballistic impact are severe problems of 2D plain fabrics, which 3D fabrics can resolve. The unique structure of 3D fabrics enhances mechanical properties along the thickness, improves structural integrity, and facilitates stress transfer between layers [109].

##### Orthogonal Interlock Structure

The stuffer, binder, and weft are three critical constituents of 3D orthogonal fabrics, which are aligned orthogonally in the horizontal, vertical, and transverse directions. The mechanical and structural characteristics of 3D fabrics can be influenced by altering the weave pattern of the yarns [110]. It is well known that stuffer yarns absorb more energy than binder yarns due to the low amount of crimp. Therefore, an optimum stuffer-to-binder yarn ratio is preferred for the best performance. Sun et al. [111] conducted a study on the orthogonal interlock structure for ballistic impact, and they found that during the ballistic study (numerical and experimental), there was no delamination in 3D woven fabric owing to the highest in-plane modulus and delamination resistance. Further, Wu et al. [73] identified the influence of clamping and weave structure on 3D orthogonal woven fabric. They found that the clamping technique has a notable influence on the impact resistance properties of the fabric. In contrast, the effect of the weave architecture on the ballistic response of multilayer 3D orthogonal fabric systems is minimal.

##### Orthogonal Angle Interlock Layer-to-Layer

Three-dimensional orthogonal interlock fabrics are also termed warp-interlock 3D fabrics. The structure consists of several layers’ warps of warp and weft yarns. Due to its typical layer arrangement, 3D warp interlock fabric exhibits significant delamination resistance, high ballistic resistance, and structural integrity [112]. Previous research also suggested that an optimum stuffer-to-binder yarn ratio offers better mechanical and structural properties than 2D plain weave fabrics [113]. Moreover, an investigation was also conducted into the impact of yarn and weave densities on the mechanical behavior of 3D warp interlock fabrics [114]. Nasrun et al. [115] studied how varying weft densities (12 to 24 picks per cm) of 100 Tex of polyester-plied yarn affect the mechanical performance of 3D angle interlock fabric. Their report showed that increasing weft density enhanced the tensile strength of 3D fabric. A comparative study on 3D warp interlock and 2D weave aramid fabric was conducted by Abtew et al. [75]. Based on the experimental results, the study concluded that ballistic performance does not significantly differ between 3D and 2D fabrics of similar density and increased layering. However, 3D fabrics exhibit better structural stability when designing body armor than 2D plain weave fabrics. Hence, the properties of a 3D warp interlock can be optimized by adjusting the stuffer-to-binder ratio, the number of layers, and yarn density, whereas the binding depth remains the sole factor that can compromise tow strength within the 3D structure.

##### Angle Interlock Through-Thickness

This architecture is called 3D angle interlock, in which warp and weft yarn cross over each other at a skewed angle. This increases the crimp angle interlock through-thickness 3D fibers compared to the angle interlock layer-to-layer structure [116]. Due to lower yarn interlacing, these structures are inferior to other 3D woven fabrics in terms of energy absorption and impact resistance. Yang et al. [117] reported that 2D fabrics with higher yarn interlacements per unit area exhibit superior energy transfer to adjacent yarns compared to 3D angle-interlock fabrics. Experimental studies by Yang et al. [118] disclosed that the impact resistance of angle-interlock fabric is comparatively lower than that of other fabrics. They developed a new 3D curved ballistic fabric combining angle-interlock and orthogonal-interlock structures to enhance ballistic performance. They found that the new 3D structure provides better ballistic resistance with the same moldability as the angled interlocked structure. Recently, Wei et al. [119] conducted a comparative study on angle-interlocked fabric and its variants using mesoscopic fabric models. They found that incorporating supplemental straight warp yarn enhanced the impact response of an angle interlock structure.

### 4.5. Influence of Environmental Conditions

Several studies have confirmed that despite the use of the best fabrication techniques, armor materials remain vulnerable to moisture, and high-temperature aging results in a high degradation mechanism [42,43]. Akay et al. [44] found that moisture absorption behavior of Kevlar 49 epoxy composite results in a significant reduction in glass transition temperature as well as mechanical properties. Moreover, moisture does not influence the elastic modulus but affects compressive, interlaminar shear and flexural strength. Arrieta et al. [120] examined the hydrolytic and photochemical aging response of Kevlar-PBI blend fabric. They found that both moisture and UV radiation significantly degrade the mechanical properties. Further, the effect of moisture on the mechanical properties of Kevlar/PVB composite with TiO_2_ nanoparticles was investigated [121]. The authors observed that both tensile and flexural properties of a Kevlar/PVB composite are lower than that of a dry sample. Therefore, it can be concluded that moisture has a significant impact on the mechanical properties of high-performance fabrics, and to control this, a fiber coating or fiber medication could be effective.

### 4.6. Influence of Projectile Geometry

**Figure 20:**
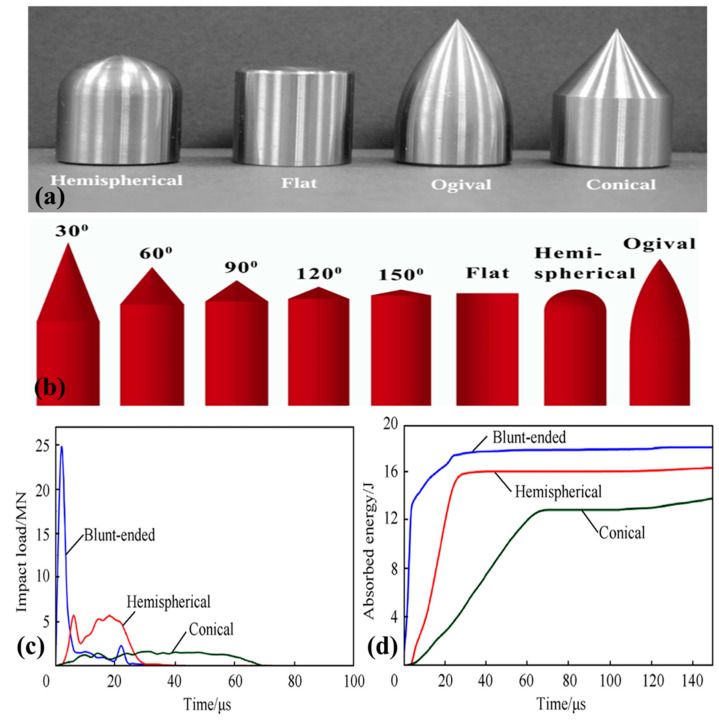
(**a**) Different projectile geometries used for perforation resistance of fabric (reprinted with permission from [122], Elsevier, 2003). (**b**) Front view of the conical projectiles with varying nose angles (reprinted from [123], MDPI, 2021). Curves of (**c**) impact load vs. time, (**d**) residual velocity vs. time at strike velocity of 176 m/s (adopted from [124], Defence Technology, 2023).

The penetration and perforation resistance of a composite against projectiles is crucial for ballistic applications. The mass, velocity, and shape of the impact significantly govern the ballistic response of a composite material, and substantial research has explored how different projectile geometries impact this performance. An investigation of the perforation resistance of synthetic fabric by projectiles with different geometries (flat, hemispherical, ogival, and conical) was performed by Tan et al. [122]. The different impactors used in this study are shown in Figure 20a [122]. Experimental results indicate ballistic limits of 159 m/s (hemispherical), 100 m/s (flat), 76 m/s (ogival), and 58 m/s (conical). Moreover, it was reported that the fabric absorbs maximum energy with hemispherical projectiles, while it absorbs the least with ogival or conical projectiles due to the better stress distribution during impact.

**Figure 21:**
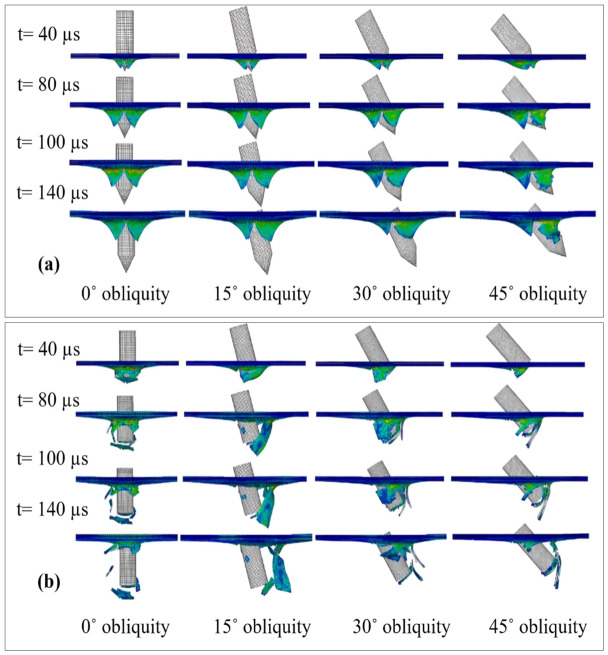
Damage progression in the laminate subjected to impact: (**a**) conical projectile and (**b**) blunt projectiles at different angles (0°, 15°, 30°, and 45°) of incidence (reprinted from [124], Defence Technology, 2023).

In the same year, Ulven et al. [125] analyzed the impact of the projectile shape on carbon/epoxy composites. Two composite panels of dissimilar thicknesses were fabricated, one consisting of 7 layers (3.2 mm), and the other having 17 layers (6.5 mm) of carbon fabric. They also found that crack growth is very significant in conical projectiles. Moreover, the panel thickness effect was found on the ballistic limit with different projectiles. In this continuation, Mitrevski et al. [126] found that conical indenters can penetrate to an immense depth and exhibit the greatest impact energy absorption compared to ogival and hemispherical indenters for different carbon/epoxy composites. Talebi et al. [127] analyzed the effect of the nose angle (30° to 180°) on the impact response of Twaron fabric via finite element modeling. Further, Shen et al. [123] also investigated the effect of varying the nose angle on the energy absorption capacity of UHMWPE fabrics. Figure 20b [123] illustrates the FEA models of varying projectile nose angles with a diameter of 15 mm and a mass of 54 g. They noticed that a small nose angle causes shear failure in the laminate at the restricted zone, while large and flat-nose projectiles cause tensile deformation in the laminate. Furthermore, the influence of conical, hemispherical, and blunt-ended impactors with obliquities of 0°, 15°, 30°, and 45° on the impact response of a woven fabric composite was investigated by Goda [124]. The study examined the impact of various projectiles on impact load, residual velocity, and energy absorption by woven fabric during impact tests. They observed that a conical projectile results in a longer contact time, lower impact force, and less fabric damage. In contrast, a blunt projectile reaches peak load abruptly, generates higher force more quickly, and causes severe damage. The study also revealed that with an increase in impact angle, the projectile takes a longer time to penetrate the laminate, resulting in greater deformation at more oblique angles, as outlined in Figure 20c,d [124]. Additionally, higher energy absorption was found with blunt projectiles due to the higher contact area compared to conical projectiles, as illustrated in Figure 21a,b [124].

## 5. Improving the Impact and Stab Resistance of Soft Armor Materials

Scientists have looked into various strategies to achieve enhanced ballistic protection without compromising overall weight. Using natural latex/rubber, hybridizing fibers with STF or nanomaterials, such as graphene or CNT, and utilizing 3D fabrics stand out among existing methods. Latex and surface modification are applied to improve the structural integrity, whereas STFs are employed for friction enhancement between yarns. The objective of using 3D fabrics aims to enhance the structural stability by positioning yarns in the Z-axis. This section highlights some of these methods.

### 5.1. Latex/Natural Rubber

**Figure 22:**
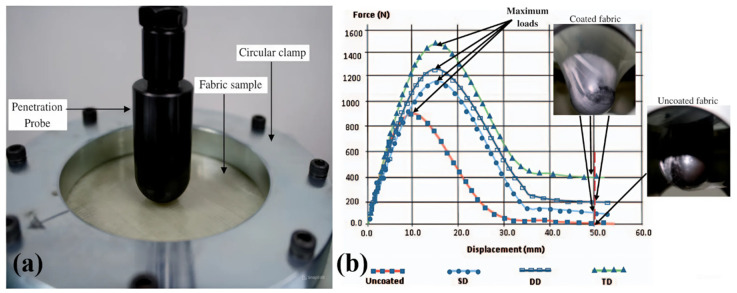
Puncture resistance test: (**a**) test setup (reprinted from [128], Sage, 2011) and (**b**) force vs. displacement curve for different rubber-coated fabrics (reprinted from [128], Sage, 2011).

The latex/natural rubber has been employed with UD high-molecular-weight polyethylene by Hassim et al. [128] to investigate the puncture resistance performance using ASTM F1342, as presented in Figure 22a [128]. The investigation revealed that natural rubber-coated fabrics can maintain fabric stability and protect the surface against damage. In addition, puncture resistances of 39%, 47%, and 62% were recorded for single-dipped (SD), double-dipped (DD), and triple-dipped (TD) fabric, respectively, compared to uncoated polyethylene fabric, as displayed in Figure 22b [128].

**Figure 23:**
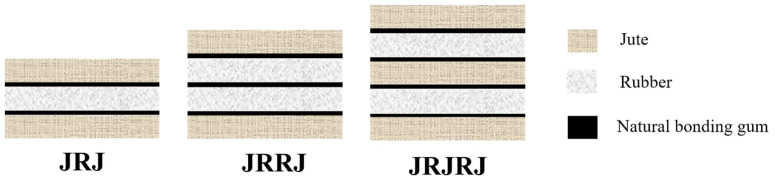
The schematic of jute rubber composites arranged in different stacking sequences (reprinted with permission from [132], Elsevier, 2019).

Furthermore, the rubber layer is integrated into a CFRP laminate to enhance impact resistance, as reported by Stelldinger et al. [129]. They noted that softer rubber compounds provide higher impact resistance, and that the position of the rubber layer influences the damage threshold load. They found that positioning a rubber layer near the outer zone increases the average damage threshold load by 31%. The high-velocity impact response of neat Kevlar and its composites with thermoset and rubber matrices was examined using a gas gun [130]. The study demonstrated that a high-hardness rubber matrix enhanced the ballistic limit by approximately 19% and 41% for two- and four-layer fabrics, respectively, compared to the neat fabric. The research was further extended to include natural fibers, particularly jute fibers and their composites [131]. Mahesh et al. [132] analyzed the low-velocity impact of jute rubber composites using various fabric arrangements, as shown in Figure 23 [132]. The outcome of the study demonstrated that jute–rubber–jute–rubber–jute (JRJRJ) exhibited better damage resistance compared to jute–rubber–rubber–jute (JRRJ) and jute–rubber–jute (JRJ) composites, but caused the impactor to bounce. On the contrary, JRJ showcased a better energy absorption capability than JRRJ and JRJRJ composites.

Similarly, Rajole et al. [133] also investigated the influence of rubber on jute epoxy composites, and the findings of the study support the previous studies and affirm the decisive role of rubber in improving the energy absorption capability of jute epoxy composites.

### 5.2. Shear Thickening Fluids

STFs refers to highly concentrated colloidal suspensions that exhibit a rapid change in viscosity when subjected to elevated shear rates. STFs are also described as intelligent, smart, or reversible fluids that behave like a solid under high-impact load and normal fluids when a gradual load is acted upon [45]. From a fluid mechanics point of view, STF is categorized as a dilatant fluid. Fluids are broadly classified into Newtonian and non-Newtonian fluids. The shear stress and shear rate are linearly associated with each other in a Newtonian fluid; conversely, the shear stress is usually nonlinear for a non-Newtonian fluid. In dilatant fluid, the shear stress increases exponentially with an increase in shear rate, while pseudoplastic fluid displays a distinct nonlinear profile [10]. The strain rates vary as a function of time for Rheopectic and Thixotropic fluids, and are subjected to some pre-shear stress. Among all non-Newtonian fluids, dilatant fluids have been investigated to develop innovative materials for various engineering applications, including dampers, sports equipment, medical devices, and protective materials. The research study unveiled the potential of STFs for improving the performance and efficiency of protective systems. In later years, these fluids were suggested as shock-absorbing materials for earthquake-resistive structures, soft-landing spacecraft gears, and medical equipment [134,135,136,137,138]. In recent times, multiphase STFs comprising additives such as ceramics and carbon-based particles in suspension have been developed to investigate their benefits for designing protective systems [139]. The shear thickening phenomenon has attracted many researchers, where mechanisms like order–disorder transition (ODT), hydrocluster theory, jamming, and dilation theory define the behavior of STFs. The proposed theories are described in the sub-sections.

#### 5.2.1. Order–Disorder Theory

ODT was introduced by Hoffman [140] in 1998. According to this theory, when a concentrated colloidal solution is acted upon by shearing forces at a low shear rate, the particles in the suspension (due to Brownian motion) prevent interaction with adjacent particles because of the inherent repulsion between them. Particles are arranged in order at low shear rates and form a stable layered arrangement. However, at an elevated shear rate, the stronger shear force outplays the repulsive particle–particle interaction beyond a certain point (critical shear rate). This results in a disruption in the ordered flow and disordered arrangement of the particles in the suspension.

#### 5.2.2. Hydro-Cluster Theory

Hydro-cluster theory, also known as particle cluster theory, was proposed by Bossis et al. [141]. They stated that ODT does not completely define the shear thickening phenomenon because particle clustering plays a vital role. Brownian motion is prevalent at a low shear rate, and particles arrange themselves in a layered form, leading to a shear-thinning effect. The suspended particles interact with each other, resulting in a rise in hydrodynamic pressure at higher speeds, and leading to non-equilibrium conditions in the fluid. With a further increase in shear rate, self-organizing microstructures, also known as particle clustering, develop, impeding fluid flow, and ultimately leading to an abrupt change in the fluid’s viscosity. The hydro-cluster theory explains continuous shear thickening (CST) in which the viscosity of the suspension continuously increases with the temporary establishment of particle clustering. Wagner and Brady [140] also confirmed the formation of hydro-clusters and sudden viscosity changes in shear-thickening fluids, as discussed in our earlier publications [142]. Further, some studies have identified the phenomenon of discontinuous shear thickening (DST) as a volume fraction surpasses a threshold value (*ϕ_c_*), which is governed by the suspended particles. CST occurs below ϕc, and the intensity weakens as the volume fraction decreases. However, the rapid change in viscosity with a growing shear rate is a typical feature in dense suspensions, valid for both Brownian and non-Brownian suspensions.

#### 5.2.3. Dilatancy Theory

Hydro-cluster theory explains the CST phenomenon; however, the theory fails to define the DST effect. Dilatancy has been associated with DST for a long time. In earlier studies, dilatancy was used as a synonym for STFs. Still, Metzner and Whitlock [143] found that although dilatancy and shear thickening typically coincide, dilatancy can sometimes occur without shear thickening in certain suspensions [144]. Dilatancy refers to the increase in volume of a dense granular flow under shear; the particles require more space to rearrange themselves, which can induce further stresses due to solid–solid friction [145]. When a fluid experiences increasing shear forces at a constant pressure, the fluid particles rearrange themselves, increasing the viscosity of the fluid or the resistance to flow. The sharp increase in viscosity is due to dilatancy, causing particle rearrangements that lead to a temporary jam-like state. The jamming network is often associated with frictional or contact forces dominating hydrodynamic forces at a high shear rate.

#### 5.2.4. Contact Rheology Model

The contact rheology model theory can address the limitations of hydro-cluster theory. According to hydro-cluster theory, the particles in the suspension interact with each other through hydrodynamic forces. However, the theory does not explain the effect of solid friction when particles come into direct contact and behave as a granular material. At a high shear rate, hydrodynamic forces become ineffective in keeping particles apart, leading to frictional contact between particles. The relative motion of particles and the pressure between them drive the transition. Seto et al. [146] proposed a new model incorporating hydrodynamic interactions and granular-like contacts. The study highlighted that friction facilitates the development of interconnected contact networks (percolation) through local particle rearrangements (dilatancy). Percolation occurs over a small range of shear rates, enabling it to exhibit unique mechanical properties. Furthermore, Qin et al. [147] presented a theoretical model to explore the thickening behavior of the suspension. The study revealed that higher particle loading in suspension reduces the spacing between particles, making it easier for them to form clusters. Moreover, more particle clusters formed at higher particle loading or shear rates, leading to the fluid being jammed in the rheometer. Therefore, this model can predict both CST and DST phenomena more accurately.

### 5.3. Parameters Influencing Shear Thickening Behavior

The particle aspect ratio, particle–particle interaction, roughness, volume fraction, pH, environmental conditions, and type of liquid medium can affect the performance of STF. The following section details the various factors that impact STF performance, both directly and indirectly.

#### 5.3.1. Particle Content

**Figure 24:**
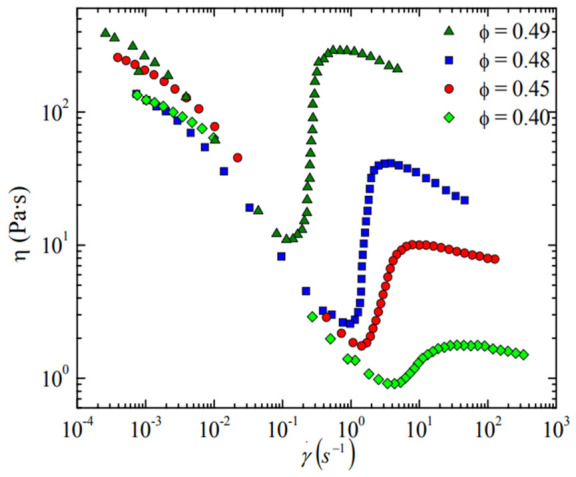
Steady rheology data of PMMA-based STF at varying ϕ (reprinted from [150], Proceedings of the International SAMPE Symposium and Exhibition, 2007).

The particle volume fraction is a fundamental parameter in the synthesis of STFs. In one of the early studies, Lee et al. [148] employed colloidal silica at a volume fraction (*ϕ*) of 0.57 and 0.62 in ethylene glycol (EG). The rheological study revealed that the shear thickening effect was evident at a shear rate of 10 s^−1^ for ϕ = 0.62 and 300 s^−1^ for ϕ = 0.57. In addition, at high shear rates, the high-volume fraction (ϕ = 0.62) suspension exhibits a significantly high viscosity. Kalman et al. [149] also investigated the role of multiple volume fractions (*ϕ* = 0.40, 0.45, 0.48, and 0.49) in improving the shear thickening effect using monodisperse poly methyl methacrylate (PMMA) in EG. They noted that the viscosity sharply increases with a higher particle volume fraction, and the shear thickening effect is observed at a lower shear rate when the volume fraction is high compared to other STFs, as presented in Figure 24 [150].

Petel et al. [151] also evaluated the response of STFs developed using cornstarch, SiC, and SiO_2_ with varying volume fractions in EG. Three suspensions were examined, where two were silica-based (61 SiO_2_, *ϕ* = 61.5%, and 61 Mix, *ϕ* = 47.6%), and the third was cornstarch-based (54 CS, *ϕ* = 47.6%). They noticed that increasing the value of *ϕ* influences the viscosity of the suspension.

#### 5.3.2. Particle Aspect Ratio and Shape

**Figure 25:**
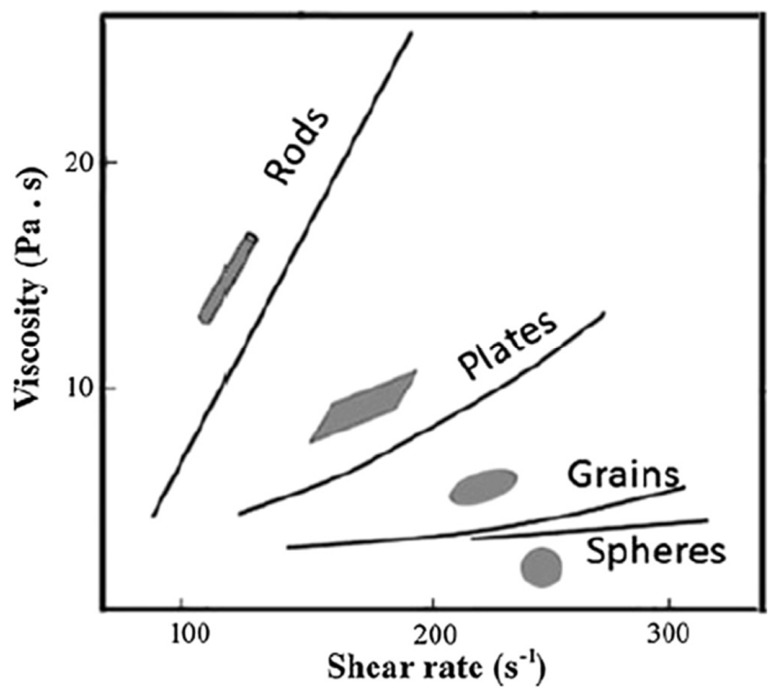
Effect of particle shapes on the viscosity of the suspension (reprinted from [153], 12th International Conference on Latest Trends in Engineering and Technology (ICLTET’2017), 2017).

The interaction of the particle aspect ratio and volume fraction considerably affects STF behavior. Research shows that the critical shear rate (CSR) reduces with an increasing aspect ratio and that a higher particle aspect ratio reduces the solid *ϕ*, essential for shear thickening [144]. The effect of particle shapes was found to be associated with the aspect ratio of particles. In early studies, Barnes et al. [152] noted that different particle shapes yielded distinct viscosity profiles. They found that cylindrical-shaped particles were the best-performing particles to strengthen the thickening effect compared to spheres, grains, and plates, as displayed in Figure 25 [153]. It is clear from Figure 25 that with rod-shaped particles, there is substantial growth in viscosity at a shear rate of 200 s^−1^, whereas in the context of spheres, the viscosity rises above a shear rate of 300 s^−1^.

Furthermore, calcium carbonate (CaCO_3_) particles with varying aspect ratios of 2:1, 4:1, and 7:1 were used in conjunction with spherical silica particles (120 nm) to assess the influence of different ratios on the thickening effect of the developed STFs by Wetzel et al. [154]. The results of this study are consistent with those of earlier studies, confirming that particles with higher aspect ratios enhance the thickening effect and reduce the CSR for CaCO_3_-based STFs.

#### 5.3.3. Particle Size and Distribution

**Figure 26:**
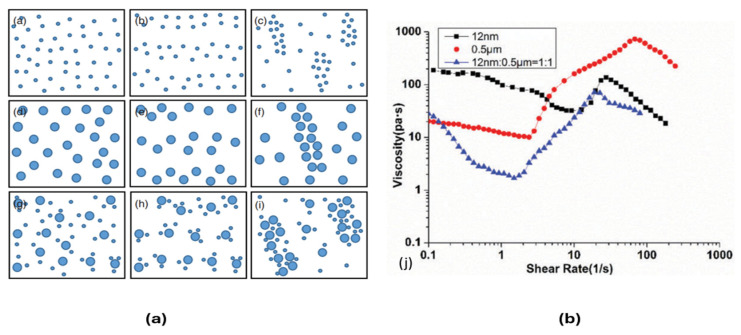
Rheology of silica colloidal suspension: (**a**) viscosity vs. shear rate for different particle sizes, (**b**) CSR vs. particle size curve (reprinted from [158], Sage, 2022).

The size of solid content is also a crucial factor affecting the thickening effect of STFs. Maranzano and Wagner [155] have investigated the contribution of the particle size to colloidal dispersions. It has been reported that the flow curves progressively shift towards lower shear stress with an increase in particle size, for a fixed volume fraction (ϕ = 0.50). Another study examined the impact of silica particles (100 nm, 300 nm, and 500 nm) in PEG-200 to develop STF [156]. The outcome established that as the particle size decreases, the CSR also reduces, even for a constant weight percentage of silica particles in PEG. The influence of five different silica particles with sizes of 15 nm, 30 nm, 2 μm, 5 μm, and 10 μm was examined for their effect on the rheological behavior of the suspension [157]. The experimental work emphasizes that, despite differences in particle size and concentration, the trend in each flow curve remains consistent. The study found that silica micro-particles lead to higher-particle-concentration fluid, exhibit higher CSRs, and offer poor stability due to longer sedimentation than silica nanoparticles. Zhang et al. [158] conducted their experimental work with 70 wt.% of PEG and three different combinations of SiO_2_. They utilized 12 nm, 0.5 µm and the combination of both for their study. The study confirmed that the blending of each particle contributes higher viscosity than the individual due to more stable hydro-cluster formation, as depicted in Figure 26 [158]. Figure 26a exhibits the particle behavior under shear (a–c) for 12 nm, (d–f) for 0.5 µm, and (g–i) for blend particles. Additionally, the authors found that large SiO_2_ shows higher peak viscosity than the smaller particle and even blended particles due to a strong clustering effect, as depicted in Figure 26b.

Recently, Lu et al. [159] also investigated the puncture resistance response of STF-impregnated fabric using different fumed silica particle sizes (12 nm and 40 nm). The outcome of the study revealed that the initial viscosity of smaller silica is higher than that of STFs made from larger silica for the same weight percentage, due to the large surface area-to-volume ratio of nanoparticles. Moreover, the study confirmed that particle size has a significant impact on the CSR, with the thickening effect occurring at lower CSR values for larger silica particles.

#### 5.3.4. Particle–Particle Interaction

Earlier observations revealed a system of particles that are either neutral or repel each other via electrostatic, entropic, or steric forces, inducing a thickening effect. Barnes et al. [152] explained that deflocculated suspensions, where the particles are well dispersed, maintain low viscosity under low shear conditions while experiencing enhanced shear thickening at elevated shear rates, resulting in rheopexy. On the other hand, a flocculated suspension, where the particles are clustered, demonstrated pronounced viscosity at a reduced shear rate; however, the suspensions showcase shear thinning at an elevated shear rate, generally showing thixotropy.

#### 5.3.5. Particle Roughness

The effect of particle roughness on concentrated colloidal and non-colloidal suspensions has been investigated [160]. The effect of a sphere of polystyrene (PSt), PMMA, and glass on the Newtonian (silicon oil) and non-Newtonian (Boger fluid) rheological behavior was studied by Moon et al. [161]. The average particle roughness levels were 93, 32, 55, and 30 nm for PMMA (40 µm), PSt (40 µm), PSt (80 µm), and glass (40 µm), respectively. The finding disclosed that particle roughness enhances the frictional resistance and particle–particle interaction, resulting in elevated viscosity. Later, Hsiao et al. [162] designed a model to find out the influence of four PMMA particles, including smooth, slightly rough (SL), medium rough (MR), and very rough (VR) particles. Atomic force microscopy images of smooth, SL, MR, and VR colloids was investigated at a scale of 1 μm. The flow curves of suspensions with *ϕ* = 0.30, 0.35, 0.40, 0.45, 0.48, 0.50, 0.535, and 0.55. The first normal stress difference (N_1_) vs. stress (σ) curves were plotted with standard deviations from three independent upward stress sweeps.

At low stress (σ), the suspension exhibits nearly constant viscosity (*η*) in the Newtonian regime. As the σ increases, η elevates sharply from the critical stress point (σ_c_), indicating the onset of thickening in the colloidal system. It was found that smooth particles exhibit a milder thickening effect than rough particles. Interestingly, σ_c_ does not vary with *ϕ* for smooth colloids but declines with increasing *ϕ* for rough particles. Additionally, the N_1_ curve for smooth colloids remains negative, whereas for rough colloids, it becomes positive at lower concentrations due to particle clustering.

#### 5.3.6. Particle Hardness

Kalman et al. [149] examined the penetration behavior of STF-treated fabrics using SiO_2_ (500 nm) and PMMA (1050 nm) particles with PEG-200, with ϕ values of 0.52 and 0.49, respectively. They observed that a harder particle-based STF-fabric (SiO_2_) is more effective at reducing yarn and filament mobility than a softer one (PMMA). They found that hard particles make intense mechanical contact with fabrics, thereby improving mechanical performance. In a later study, Petel et al. [151] used cornstarch, SiO_2_, and SiC particles with hardness values of n/a, 8.3, and 30.8 GPa, respectively, along with EG to prepare STFs and subsequently analyzed their ballistic resistance. They noted that the Mix (SiO_2_ and SiC) STF and the SiO_2_-based STF exhibited the best penetration resistance at ϕ = 0.62 over impact velocities between 200 and 700 m/s. The cornstarch-based STF with *ϕ* = 0.54 exhibited poor ballistic penetration resistance due to its lower yield strength compared to the other two.

#### 5.3.7. Effect of pH

Titanium dioxide (TiO_2_) and Millipore-filtered water suspensions were investigated by Mikulasek et al. [163] for the effect of pH in the presence of sodium hexametaphosphate. The viscosity was examined at a shear rate of 500 s^−1^ for 1, 10, 30, and 50 vol% TiO_2_ in water. Within the basic region of pH 7 to 14, the apparent viscosity shows an initial reduction with pH, hits a minimum at pH 9, and then increases further. On the acidic side (pH 7–0), the same trends were observed. The authors found that as pH decreases, the suspension’s ionic strength increases, leading to flocculation attributed to van der Waals forces. Furthermore, Chen et al. [164] examined the role of pH in determining the STF response of colloidal dispersions, utilizing polystyrene-ethyl acrylate (PSt-EA, 400 nm) dispersed in EG. Different solutions, including hydrochloric acid (HCl), citric acid, sodium citrate, sodium acetate, sodium hydroxide (NaOH), and potassium hydroxide (KOH), were prepared and added to the suspension to investigate the effect of pH. The study concluded that pH values substantially influence shear-thickening behavior and that the CSR declines with a drop in pH when acid is added; however, the opposite is observed with alkali.

#### 5.3.8. Effect of Temperature

The influence of temperature on the dispersion of PS particles in a glycerol/water mixture (86.1/13.9, *w*/*w*), with a volume fraction of 0.57, was examined by Boersma et al. [165] at various temperatures (20–50 °C). The study confirmed that viscosity decreases with increasing temperature. In another study, Mewis and Biebaut [166] conducted an extensive rheological analysis using silica particles coated with poly-butyl methacrylate as a suspending medium in octanol with m-xylene. Viscosity curves at different temperatures for two volume fractions (ϕ = 0.315 and 0.404) were plotted. A more concentrated suspension results in higher viscosity at high temperatures at a low shear rate, but a drop in viscosity is reported at a higher shear rate. Progressively, Tain et al. [167] also confirmed that at high temperatures, the suspension leads to low viscosity, higher CSRs, and reduced shear thickening effects during experimental work with 20 wt.% fumed silica-loaded EG-based STFs. Moreover, the viscosity vs. shear stress was also studied in relation to temperature. They found that the viscosity initially drops and then increases, eventually dropping again with an increase in shear stress for all STFs, but the viscosity decreases with an increase in temperature. Warren et al. [168] and Liu et al. [169] further validated the same outcomes for fumed SiO_2_-STFs.

#### 5.3.9. Effect of Liquid/Carrier Medium

**Figure 27:**
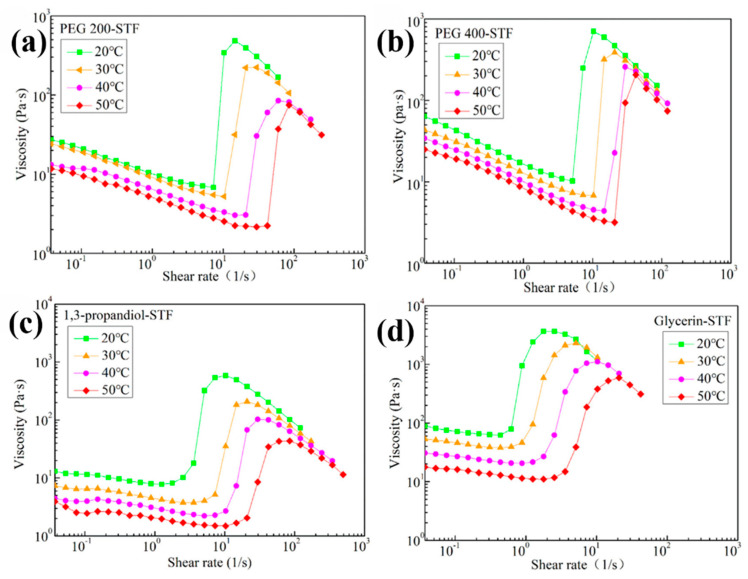
Rheological behavior of STF under varying temperatures: (**a**) PEG 200-STF, (**b**) PEG 400-STF, (**c**) 1,3-propandiol-STF, (**d**) glycerin-STF (reprinted with permission from [164], Elsevier, 2020).

An STF is composed of solid content and a dispersion (liquid/carrier) medium. The dispersion medium contributes to the rheological properties of STFs. The effect of a varying molecular weight (20, 200, and 1000) of phenyl tri-methicone at a constant temperature and ϕ = 0.5 was investigated for the rheological performance of STF by Shenoy and Wagner [163]. Their research indicated that the high molecular weight of the dispersion medium results in elevated viscosity; however, the extent of shear thickening decreases with an increasing molecular weight. Furthermore, the STFs were prepared using SiO_2_ and CaCO_3_ dispersed in PEG-200 and 400 and assessed for rheological performance over a shear rate range of 0.1 to 1000 s^−1^ [170]. The findings revealed that both CaCO_3_-STF and SiO_2_-STF exhibit different behavior at varying shear rates. With an increasing shear rate, the viscosity of CaCO_3_-STF decreases consistently; however, clear evidence of shear thickening was observed with SiO_2_-STF. In addition, rheological trends are evident, showing that a greater molecular weight of the medium leads to a sudden change in viscosity, even at a low CSR. In recent work, four different dispersion media—namely, PEG-200, PEG-400, 1,3-propanediol, and glycerin—were used with fumed silica to synthesize STFs [164]. According to the findings, higher temperatures lead to a deterioration in the rheological behavior of STFs. The effect of temperature concerning different dispersion media is shown in Figure 27 [164]. The peak viscosity is affected at elevated temperatures (20–50 °C) for all four STFs. The glycerin-based STF showed the highest peak viscosity at 20 °C compared to other STFs due to the large number of hydroxyl groups that provided high affinity with fumed silica. However, at rising temperatures, both PEG 400-based and glycerin-based STFs performed better than the others.

#### 5.3.10. Effect of Additives

Additives such as graphene, CNTs, cellulose nanofibers, and SiC have been shown to enhance the performance of STFs. Sha et al. [165] employed carbon nanofillers (diameter: 10–20 nm and length: 5–15 µm) and graphene nanoplatelets (GNs, diameter: 50 nm and length: 20 µm) blended with silica nanoparticles (650 nm) and PEG 200 to prepare STFs. The study investigated the effect of varying additive weight percentages on the rheological performance of a 75 wt.% silica-PEG suspension. The outcome indicated that the rheological performance of STF is significantly improved with CNT and GN nanofillers. However, due to its distinct tubular structure, CNT-based STF demonstrated a superior rheological performance over GNs at identical mass fractions. In further studies, Gurgen et al. [171] assessed the impact of SiC particles with varying sizes on the rheological behavior of fumed silica-PEG 200 suspensions. The experiments showed a noticeable, consistent increase in the initial viscosity at all temperatures and particle sizes as the concentration of additives in the suspension increased. Moreover, additives with finer particle sizes were more effective in providing a high initial viscosity than those with larger particles. There was an influence of MWCNTs (diameter: 10–20 nm and length: 30 µm) on the rheological properties of the SiO_2_-STF suspension [172]. The study demonstrated that shear thickening is less pronounced with the incorporation of MWNTs, resulting in an STF with 44 wt.% silica nanoparticles.

**Figure 28:**
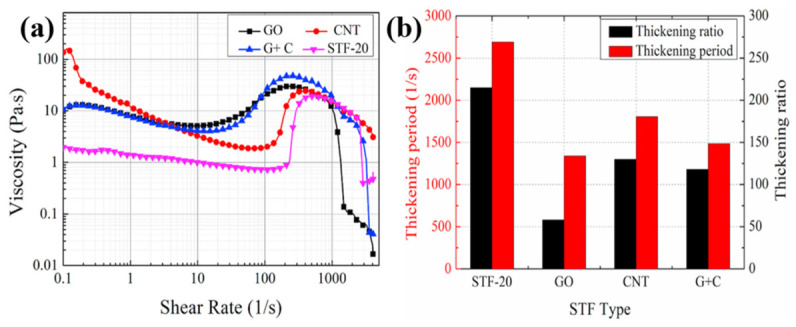
Rheological behavior of multiphase STFs: (**a**) viscosity vs. shear rate and (**b**) thickening period and ratio for different STFs (reprinted with permission from [173], Elsevier, 2020).

Recently, Liu et al. [173] developed multi-phase STFs using GO and CNT in silica-based STFs and investigated their ballistic impact performance. They found that adding GO and CNT to SiO_2_-STF increased the initial viscosity due to the higher solid content, as shown in Figure 28a [174]. Additionally, the higher aspect ratios of GO and CNT compared to spherical silica particles contributed to greater viscosity through particle interlocking. The thickening ratio of SiO_2_-STF reached 21.5 but decreased to 5.8–13.0 for multi-phase STFs due to strong interactions between silica particles and GO/CNT, leading to increased aggregation, as indicated in Figure 28b [173].

The shear thickening response of STFs is governed by a complex interplay of particle characteristics, suspension chemistry, and external conditions. Parameters such as particle volume fraction, size, shape, roughness, hardness, and inter-particle interactions critically influence the onset and intensity of thickening behavior. In parallel, environmental factors, including pH, temperature, and the nature of the carrier medium, significantly modulate rheological stability and critical shear conditions. The incorporation of functional additives further alters flow behavior through enhanced frictional contacts and particle networking. Collectively, these studies highlight that STF performance is highly tunable but strongly system-dependent, underscoring the need for application-specific design strategies. This understanding provides a robust foundation for evaluating current limitations and identifying future research directions.

## 6. Impact Standards and Testing

Body armor is evaluated using specific testing standards that assess various types of bullets differing in shape, size, and materials. The delivery of bullets to the target, from handguns, rifles, machine guns, and snipers, also influences the performance of armor material when subjected to ballistic impact [175]. Military Standard 662 Revision F (MIL-STD 662F), Standardization Agreement (SATNAG-2920), NIJ, the UK Home Office Scientific Development Branch (HOSDB) and GJB are among the prevalent standards for ballistic impacts generally employed by researchers. Several sharp and pointed objects are found in life-threatening injuries to army personnel, causing multiple cuts, slashes, and piercings [7]. Such objects fall into two categories: domestic or utility knives and spike tools, including screwdrivers. These objects are further differentiated by geometry and various nose angles.

### 6.1. Ballistic Resistance Standards

Five necessary ballistic resistance standards, MIL-STD 662F, SATNAG, NIJ, HOSDB and Chinese, are generally followed when designing body armor, as discussed below.

#### 6.1.1. Military Standard 662 Revision F

All departments and agencies of the Department of Defense, USA, approved this standard for use on 18 December 1997 [176]. This standard provides general guidelines for determining the V50 ballistic limit of armor materials against projectiles. As specified by the standard V50, the mean of the upper partial-penetration velocity and the lower total-penetration velocity in the test range is calculated. The testing procedure involves using specified types of calibers with listed velocities, as described in the standard. Chronographs measure the velocities of projectiles, and sometimes, Doppler radar is also used to enhance the reliability of the measured velocity and provide additional validation. The test sample is placed perpendicular to the projectile, which can be adjusted vertically, horizontally, and obliquely at an oblique angle. All ballistics tests are preferred to be conducted in standard atmospheric conditions of 23 ± 2 °C and with a relative humidity of 50 ± 5%.

#### 6.1.2. Standardization Agreement

The SATNAG-2920 standard was introduced on 31 July 2003 by the North Atlantic Treaty Organization (NATO). The standard provided ballistic test methods for personal armor materials [177]. The agreement aims to establish testing guidelines for ballistic threats posed by projectiles, bullets, and flechettes. The specific category of this agreement is designed to cover the protection guidelines for helmet shell, face, and eye protection [178].

**Guideline for test equipment and procedure**.

**Projectiles:** Bullets, flechettes, fragment simulators, or any ballistic projectile that could pose a potential threat to personnel.

**Fragment simulators** are defined by their mass and length. The mass, length, and diameter of all fragment simulators are described in the agreement. It generally includes steel cylinders, spheres, cubes, and parallelepipeds.

**Velocity range:** The ballistic limit is anticipated to fall within an average velocity of 80 ± 15 m/s.

**Armor size and clamping:** The sample materials should be tightly affixed to the rigid framework in a direction perpendicular to the armor surface.

#### 6.1.3. National Institute of Justice

NIJ is a prominent benchmark for evaluating the impact performance of soft body armor, introduced by the US Department of Justice in 1972 with NIJ-0101.01 [179]. The document provides guidelines for the minimum ballistic resistance performance required of armor against ballistic impact. The ballistic performance of soft body armor is segmented into five classes (IIA, II, IIIA, III, and IV). The caliber type, ammunition type, specific mass, minimum velocity, and BFS are summarized in Table 8.

NIJ 0101.06 is intended to evaluate the ballistic limit by recording the velocities using chronographs. V50 is a different way to access the ballistic limit of the armor panel, defined as velocity corresponding to a penetration probability of 50%.

#### 6.1.4. UK Home Office Scientific Development Branch

HOSDB published stab-resistant body armor test specifications in 1993 and 1995, and later the ballistic body armor standard in 1995. The standard provides seven different levels of protection, as outlined in Table 9 [180].

#### 6.1.5. Chinese Protection Standards

Chinese GJB 150.11A-2009 is for laboratory environmental test methods for military materials. The purpose of this test is intended to determine the effectiveness of the protection system. Additionally, the test gives an idea of corrosion, electrical, and physical effects.

### 6.2. Stab Resistance Standards

Stab-resistant armor is evaluated for compliance with the VPAM (Germany), HOSDB (UK), and NIJ-0115.0 (USA) standards. The international acceptance of the NIJ standard makes it the most widely adopted [181,182]. The purpose of NIJ-0115.0 is to establish guidelines for stab resistance of personal body armor against threats posed by knives and pointed instruments. The armors are classified into three protection levels. Level 1 is suitable for low-level protection, Level 2 is ideal for general duty garments, and Level 3 is helpful for high-risk situations [183]. All three protection levels are summarized in Table 10.

**Figure 29:**
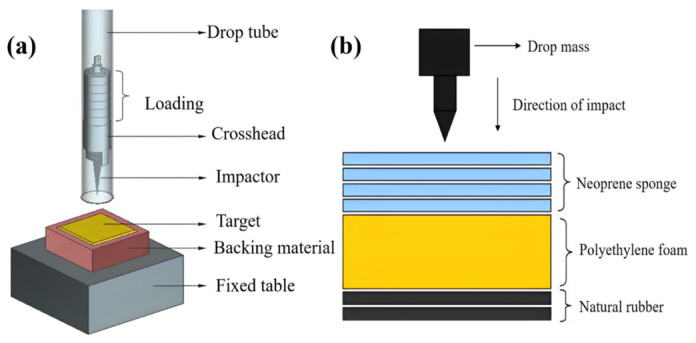
Stab resistance test: (**a**) test setup (reprinted with permission from [28], Elsevier, 2017) and (**b**) backing materials (adapted from [184], Sage, 2018).

A stabbing-resistant test is performed with a knife and spike indenter mounted on the crosshead in the rail-guided drop tower. During the stab resistance test, the indenter with a crosshead is dropped onto the armor panel and set on a backing material, including witness paper, neoprene sponge, polyethylene foam, and natural rubber sheets. The thickness of each sheet is defined in the NIJ 0115.0 standard, as depicted in Figure 29a,b [28,184]. Additionally, the penetration depth into the target is quantified by the amount of witness paper pierced by the impactor.

## 7. Performance Evaluation of Armor Material

Various testing methods are employed to assess armor materials’ ability to withstand mechanical and ballistic loads and evaluate their effectiveness in real-world applications. This section highlights the standard tests used to determine the performance of armor materials.

### 7.1. Yarn–Yarn Friction

**Figure 30:**
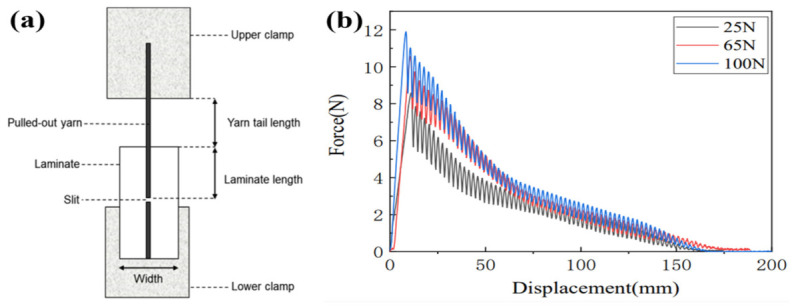
Single yarn pull-out sample: (**a**) schematic of the sample (reprinted from [185], MDPI, 2024) and (**b**) pull-out force vs. displacement plot (reprinted from [190], IOP Publishing, 2022).

The impact response of soft armor is influenced by numerous factors, among which yarn pull-out is particularly significant given the woven fabric construction. Previous studies have linked the ballistic response of woven fabrics to the number of yarns extracted during the yarn pull-out test, which is mainly influenced by inter-yarn frictional resistance, as shown in Figure 30a [185]. It is well established that impact load is predominantly concentrated on primary yarns. When the inter-yarn resistance is low, the damage occurs in the primary yarn at early stages. In some instances, it was also observed that inter-yarn friction supports secondary yarn during impact and restricts the movement of the yarns. Therefore, yarn-to-yarn friction directly influences the impact and ballistic response of woven fabrics in the design of soft armor. Nilakantan et al. [186] investigated the single-yarn pull-out test of Kevlar fabrics and observed that the pull-out load decreases with increasing pull-out speeds. The study demonstrated that pre-weaving yarn sizes and post-weaving scouring processes influence stick–slip behavior. Previous studies have shown improvements in the inter-yarn resistance of fabrics against impact loads [187]. STFs have been explored as a promising material for enhancing the impact response of woven fabrics. In one of the experimental works, p-aramid and UHMWPE fabric were treated with nano-silica-based STF [188]. The results demonstrated that the pull-out force is most evident in plain weave, at almost 4.4 times that of satin fiber, and that yarn pull-out is a key mode of fabric failure during impact. Moreover, during low-velocity impact testing, the pull-out load correlated well with the energy absorbed by the pristine p-aramid and UHMWPE fabrics and their STF counterparts. The efforts were further advanced by Khodadadi et al. [189] in their experimental work on the ballistic behavior of STF-impregnated Kevlar fabrics. Kevlar fabrics were impregnated with 15, 25, 35, and 45 wt.% of STF to assess the contribution of friction between Kevlar yarns. It was observed that inter-yarn friction increases with particle loading in STF, resulting in higher pull-out loads. The role of multi-phase STF-impregnated Twaron fabric was examined further [189]. It was noted that when nano silica (20 weight percent (wt.%)) and silicon carbide (45 wt.%) are blended with PEG-400 to form M-STF, the pullout force is significantly higher than that of single-phase (20 wt.% in PEG) S-STF and the neat fabric. Figure 30b [190] shows pull-out force vs. displacement at various loads.

### 7.2. Puncture Test

**Figure 31:**
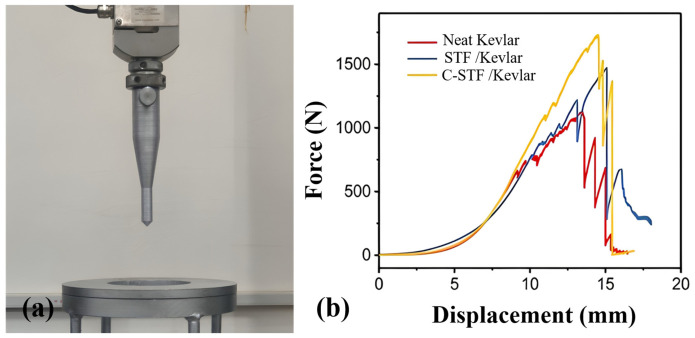
The quasi-static puncture test: (**a**) fabric subjected to the puncture test and (**b**) force vs. displacement curve (reprinted with permission from [192], Elsevier, 2020).

The puncture resistance test assesses the structural integrity of armor fabrics, protective gear, and industrial materials. The test measures a material’s ability to withstand pointed or sharp objects, as shown in Figure 31a. Impregnating woven fabric with STFs improves its puncture resistance. Baharvandi et al. [191] examined the influence of varying wt.% of silica particles blended into a PEG-based STF on the impregnation of Twaron fabric to enhance puncture resistance. They used ASTM D 6264 to evaluate the quasi-static puncture response of neat and STF-impregnated fabrics. The study found a 362% increase in puncture resistance for 35 wt.% STF-treated Twaron fabric related to the neat fabric. Furthermore, the influence of STF, developed through the combined effect of MWCNT (30 µm, 10–20 nm) and fumed silica (~12 nm), blended with PEG-200, on the impregnation of high-modulus polypropylene (HMPP) fabric was investigated [172]. The study validated that impregnating HMPP fabric with STF enhanced the maximum puncture load and energy absorption. However, due to a limited shear-thickening phenomenon, the treatment of fabric with the MWCNT-containing suspension resulted in a diminished enhancement in puncture behavior. The effect of inter-yarn friction resistance through a quasi-static puncture test on polystyrene ethyl acrylate (PSt-EA)-based STF/Kevlar composites and the CNT-doped STF (C-STF)/Kevlar composite was investigated [192]. Based on observation, the puncture process consists of two parts; in the first part, the force rises gradually, and in the second stage, the contact force exhibits a steep increase with displacement, as depicted in Figure 31b [192]. The findings revealed that STF (53.5 wt.% PSt-EA)/Kevlar showed a greater penetration force, whereas C-STF (53.5 wt.% PSt-EA and 1 wt.% CNT)/Kevlar exhibited better puncture resistance than neat Kevlar.

Recently, the scope of STF, made from recycled waste glass particles and PEG-400, was used to impregnate the naturally driven fabric reported by Chamola et al. [193]. They also confirmed the potential of STF in enhancing the puncture resistance of both single- and double-layer STF/jute fabrics. The highest puncture load was 60.42% higher for single-STF (70 wt.% MGB)/jute fabric than for the neat fabric. In contrast, a 34% higher peak load was recorded for the double-layer STF-treated jute fabric compared to the neat fabric with the same configuration.

### 7.3. Stab and Spike Test

**Figure 32:**
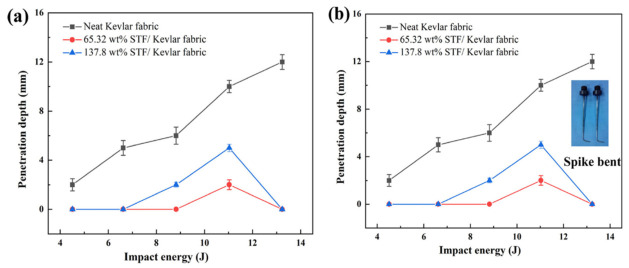
Penetration depth: (**a**) knife and (**b**) spike on the backing for different targets (reprinted with permission from [198], Elsevier, 2021).

The demand for protective materials has grown around the globe with the rising cases of violence involving knives and spikes. Protecting materials with higher stab, spike, and puncture resistance is a key engineering application in defense, safety wear, and tamperproof packaging. Moreover, such materials are necessary for daily or industrial work, where the risk of encountering sharp objects, such as pointed scraps and hypodermic needles, is high [194]. Among all the research articles, stab and spike tests are commonly performed on high-performance fabrics impregnated with STF to enhance their impact resistance properties [195]. The stab resistance of STF-impregnated aramid fabric containing 12 nm and 650 nm silica at varying concentrations (20, 25, and 30 wt.%) in PEG-200 was investigated [196]. The results indicated that the 12-layer impregnated fabric absorbed at least 58% of the impact energy, while the untreated fabric absorbed only 20%. In addition, the same areal density 12-layer panel outperformed the 24-layer untreated panel. Similarly, Li et al. [197] examined the stab performance of SiO_2_-based STF-impregnated UHMWPE and Kevlar fabrics. Interestingly, the outcomes revealed that STF/UHMWPE composite fabrics exhibited higher knife-stab resistance, whereas STF/Kevlar composite fabrics exhibited higher spike-puncture resistance. The optimal stab and spike resistance was observed for SiO_2_ particles at 25 wt.%. Further, Shang et al. [198] also assessed the stab resistance of Kevlar fabric composites reinforced with 65.32 wt.% to 137.8 wt.% by STF made of 2 µm monodisperse PSt microspheres. The fabric exhibited enhanced stab resistance, with STF values 1.5 and 5 times higher for the knife and spike, respectively. Additionally, the 137.8 wt.% STF-treated fabric has demonstrated a lower penetration depth and stronger stabbing resistance. The penetration depth of neat Kevlar fabric was reported to be 5 mm at an impact energy of 6.6 J, which was lower than 65.32 wt.% at the same impact energy, as shown in Figure 32a,b [198].

### 7.4. Ballistic Test

**Figure 33:**
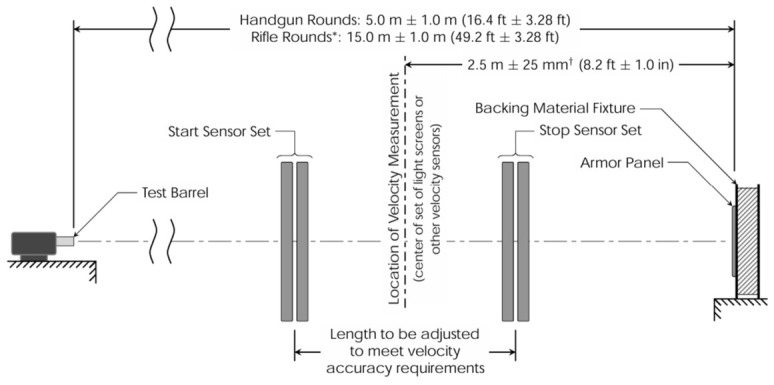
Schematic setup for ballistic test (NIJ Standard 0101.06) (reprinted from [179], US Department of Justice, 2008).

The real-life feasibility of body armor is evaluated through ballistics tests. The NIJ 0106.06 standard is generally adopted to assess the ballistic characteristics of soft body armor [179]. The ballistic test setup consists of test barrels, a sample mounting frame with backing material, and a set of chronographs equipped with optical screens to quantify projectile velocity. The screens are arranged at the recommended distance specified by the standard. The layout of the ballistic test setup is displayed in Figure 33 [179]. The armor panel is mounted at 5.0 ± 1.0 m from the muzzle of the test barrel for handguns, while the same distance is kept at 15 ± 1.0 m for rifle rounds. Moreover, the distance can be adjusted to reduce the possibility of yaw during impact; however, it should not exceed 4 m. At least two sets of velocity-measuring sensors determine the velocity of a bullet. The armor panel to be tested is held firmly in place by a backing material assembly.

**Figure 34:**
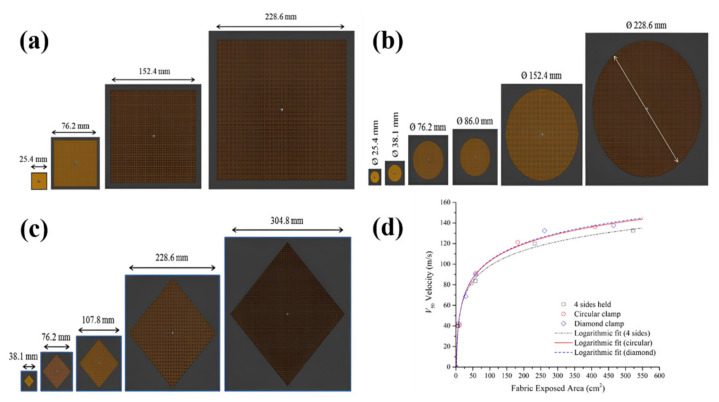
Varying sizes of targets and clamping patterns: (**a**) square clamping pattern, (**b**) circular clamping, (**c**) diamond pattern, and (**d**) fabric exposed area in different clamping pattern(reprinted with permission from [200], Elsevier, 2014).

Over time, the investigation has concentrated on determining the ballistic response of armor panels, including impact velocity, panel-to-impact distance, boundary conditions, and obliquity. Bobbili et al. [199] disclosed the effects of different configurations, thicknesses, and impact velocities on the residual velocity of a projectile of weldox steel plates against a 7.62 mm projectile using the Taguchi method. The experiment was conducted for three configurations: monolithic, double-layer, and triple-layer, with varying thicknesses of 12, 16, and 20 mm, under impact at 800 m/s and 950 m/s. It was found that the impact velocity and target thickness influence the residual velocity during ballistics tests. The effect of target geometry on the V50 behavior of soft body armor was investigated by Nilakantan et al. [200]. This study investigated targets of varying sizes for four-side clamps, circular clamps, and diamond clamping. It was observed that circular and diamond-clamped fabrics have identical V50 velocities across different fabric areas compared with the four-side clamped fabric. Furthermore, the influence of four-side and two-side clamping of aramid fabric using LS-DYNA was investigated by Nilakantan et al. [200]. The study suggested that clamping has a strong contrast with ballistic impact. They found that two-side clamping offered better energy absorption due to partially free edges, which assists in momentum transfer over time during impact. The clamping of fabric using LS-DYNA is depicted in Figure 34 [200].

**Figure 35:**
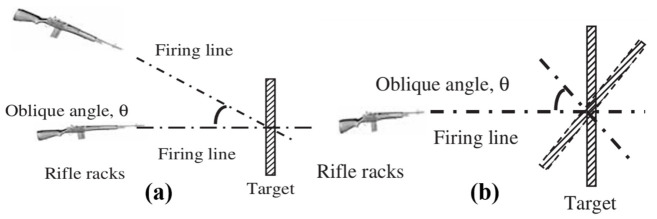
Change in obliquity: (**a**) by changing the firing line and (**b**) changing the angle of the impact surface (reprinted with permission from [2], Elsevier, 2019).

The influence of obliquity (0°, 7.5°, 15°, 30°, and 45°) on the ballistic response of Twaron and Spectra fabrics was investigated by Chu et al. [201]. The study demonstrated obliquity in two ways, as displayed in Figure 35a,b [2]. The aramid laminates exhibit a higher ricochet angle (>75°) than the metallic plate (60°) during experimental work.

Shim et al. [202] used a special fixture to hold the sample at various inclination angles. Twaron and Spectra fabrics were employed in this study, and it was found that both fabrics exhibit different behavior under obliquity. For Twaron fabric, the ballistic limit initially decreases and then increases with target inclination, whereas for Spectra fabric, it initially increases and then decreases. Moreover, the two fabrics differ in their energy-absorption spectra: the shield demonstrates greater energy absorption under angled impacts than under perpendicular impacts, whereas the Twaron fabric shows the opposite. The influence of various boundary conditions, including frame dimensions, configurations, and clamping pressures, on the impact response of soft armor was investigated by Zhang et al. [203]. It was numerically established that deformations of the armor would diminish with an increase in the value of a/r, where a is half the inner side of the frame and r is the radius of the bullet. Additionally, it was noted that increased clamping pressure reduces the bullet’s kinetic energy upon impact. The ballistic test standard highlights two important parameters: V50 and BFS, which are explored in more depth in the next sections.

### 7.5. Ballistic Limit Velocity

The ballistic limit velocity (V50) represents the mean impact speed at which half of the projectiles cause penetration, and the remaining 50% is partial penetration [8,204,205]. It is also denoted as the velocity at which the penetration probability reaches 50%. Abott and Stein [206] reported that V50 can be estimated by averaging the most significant partial and least complete penetration velocities within a desired range. The ballistic limit velocity helps quantify the ballistic performance of protective gear using US standards, such as MIL-STD-662F and NIJ-0101.06. The NIJ standard has been applied in many protection performance studies.

### 7.6. Back Face Signature

The BFS assesses blunt trauma at the rear side of the body armor on the impacted surface. It is an essential parameter for evaluating armor performance because even if the armor panel prevents bullet perforation, the transverse deflection may still be significant and fatal to the wearer. The maximum allowable BFS is 44 mm, per the NIJ 0106.01 standard, measured using standard backing material. The Roma Pastilina clay is suggested for evaluating blunt trauma, as its hardness is similar to that of human tissues or lethal organs. The Standard recommended calibrating the clay for requisite hardness before the test. Generally, the indentation depth and trauma volume are evaluated to define the BFS of an armor panel. Fahool et al. [207] evaluated the energy absorption capacity of STF-impregnated fabrics using poly-aramid Twaron fabric and flexible foam as reinforcement material. The study’s results showed that the penetration depth in BFS on plasticine is reduced in STF-impregnated fabrics compared to neat fabrics. Gurgen et al. [208] disclosed the energy transfer to the back side by measuring the trauma volume in their experimental work on aramid fabric impregnated with multi-phase (silica and silicon carbide) STF. The outcome demonstrated that multi-phase STFs provide advanced protection, significantly reducing the depth of trauma. Moreover, as the concentration of the silicon carbide additives increases, the depth of trauma reduces. Furthermore, Bajya et al. [209] conducted ballistics tests on STF/Kevlar fabrics, where STF was prepared with 500 nm and 100 nm silica particles dispersed in PEG-200, resulting in an improvement in BFS by 2.5–2.8 mm without increasing the panel weight (5 kg/m^2^). They also reported that positioning the STF/Kevlar fabric at the back and neat fabric at the striking face reduced areal density by 10% (4.5 kg/m^2^) while maintaining lower BFS.

## 8. Structural Mechanisms of Armor Panel Under Impact

The energy absorption in armor panels is highly influenced by various factors, involving material properties, projectile geometry, and their interactions over time, as discussed in previous sections. Numerous studies have explored multiple energy-absorption mechanisms in fabrics and the behavior of their composites under low- and high-velocity impacts. In low-velocity impact (1–10 ms^−1^), the contact duration of the projectile and material is long enough to respond to the impact. Hence, more energy is absorbed elastically, whereas in high-velocity (a few km·s^−1^) impact, the response is primarily governed by stress wave propagation [209]. During low-speed impact, energy is absorbed by the fabric through yarn pull-out, yarn stretching, and failure processes. In high-velocity impact, energy absorption is influenced by various factors, including fabric stretching due to pyramid or cone formation, yarn shearing, stress wave distribution, and heat generation [38,210]. Taraghi et al. [211] noticed that the energy absorption during low-velocity impact of Kevlar/epoxy can be improved by incorporating MWCNT at ambient temperature. Bandaru et al. [212] pointed out that energy absorption and impact resistance are prominently controlled by the yarns in the thickness direction and in-plane stiffness, respectively, during low-velocity impact tests (4 m/s and 6 m/s) of Kevlar/polypropylene composites. Similarly, Rheman et al. [213] noted that the composite’s energy absorption is inversely proportional to the damaged area, which can be modified by adding nanoclay, as demonstrated in their work on the low-velocity impact response of Kevlar composites. Furthermore, several studies have focused on the factors that affect the high-velocity behavior of fibrous composites. Clifton et al. [214] emphasize the importance of hybridization in the high-energy absorption capabilities of polymer composites. Thus, it is clear that the energy absorption process is influenced by velocity and the nature of the impact. Fibrous structures absorb energy through deformation, yarn extraction, rupture, fibrillation, friction, and bowing. The following mechanisms are briefly discussed below.

### 8.1. Yarn Pull-Out

**Figure 36:**
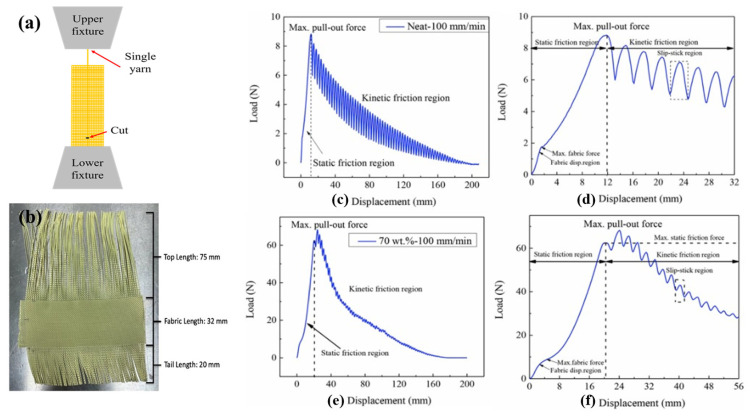
Sample preparation for yarn extraction: (**a**) single yarn and (**b**) multi-yarn, and load–displacement curves of (reprinted from [48], MDPI, 2023) (**c**) neat fabric, (**d**) stick–slip region in neat fabric, (**e**) 70 wt.% STF-impregnated fabric, and (**f**) stick–slip region for STF-impregnated fabric (reprinted with permission from [216], Elsevier, 2019).

Energy absorption during impact loading is significantly affected by friction. In woven fabric, friction occurs at the interfaces of yarns, filament–filament, and projectile yarns when subjected to high-impact loading. The fabric can achieve better friction resistance through several practices, such as stitching, rubber coating, resin application with filler materials, plasma treatment, and STFs [187,215]. The high inter-yarn friction resists lateral deformation of the woven fabric and ultimately improves fabric stability under impact loads. In addition, high frictional resistance not only restrains fabric movement but also enhances the fabric’s impact response. The yarn–yarn frictional resistance of fabric was evaluated by pulling single or multiple yarn fabric samples based on density using a UTM. The sample preparation for the pull-out test of Kevlar fabric is depicted in Figure 36a,b [48]. Analysis of the yarn pull-out test found that the stick–slip behavior of the force is observed for the yarn displacement. In their experimental work, Bai et al. [216] pointed out the effect of STF impregnation on yarn extraction experiments. They disclosed that for neat fabric, the load–displacement curve comprises two phases: linear static friction followed by oscillating dynamic friction. In linear static friction, the load increases linearly with displacement and attains a peak value, whereas it decreases gradually in a stick–slip manner due to yarn slippage at intersection points, as shown by the dashed rectangle in Figure 36c,d [216]. The curve follows a similar pattern but clearly has a seven times greater pull-out load for 70 wt.% STF-impregnated fabric compared to neat fabric, as depicted in Figure 36e,f. This indicates that STF increases inter-yarn resistance, leading to a high pulling load.

### 8.2. Yarn Failure and Fibrillation

Fibrillation is the breakdown of a single filament of high-performance fabric into small-scale fiber units called fibrils or thin strands for impact applications. Yarn fibrillation is a well-known issue in high-performance fabric, which generally occurs due to overstretching of the highly oriented fabric structure [217]. Fibrillation was investigated by Carr D. J et al. [218] in their experimental work on the failure response of yarn under ballistic impact. Later, Tan et al. [122] further investigated the perforation behavior of Twaron fabric with different geometries in an experimental study. Further, fibrillation of aramid fabric was confirmed by Lim et al. [219], and they stated that the primary failure mechanism of the transversely compressed fiber was linked to fibrillation. The influence of unprocessed aramid (virgin fabric) and plain-woven fabrics on the mechanical response was examined under high-speed loading by Tapie et al. [220]. They found that virgin fiber surfaces are intact in SEM micrographs with little fibrillation; however, micrographs of the woven fabric suggest that weaving affects their cohesion due to fibril separation and ultimately failure.

### 8.3. Pyramid Formation

**Figure 37:**
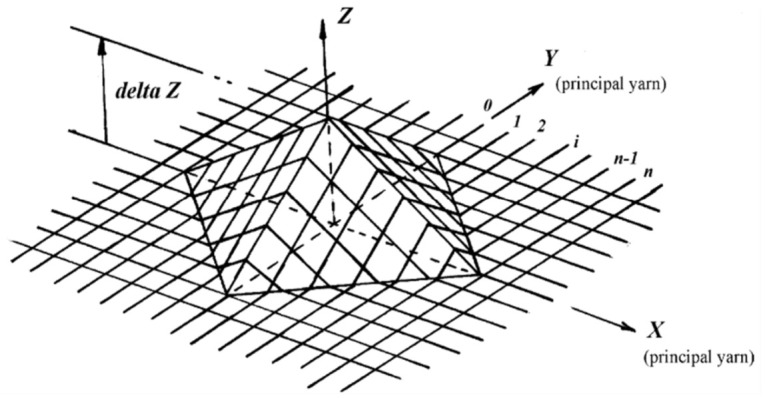
Pyramid formation in fabric under impact load (reprinted with permission from [221], Elsevier, 2003).

The longitudinal (in-plane) and transverse (out-of-plane) waves are generated at the impact point and radiate in multiple directions when a fiber is subjected to impact loads [221]. The longitudinal or strain waves originate from the impact region and travel along the warp and fill yarns at sonic speed. The transverse wave travels at a reduced speed, initiating the wave propagation occurring normal to the plane of the fabric and deformation [222]. The deformation by the transverse wave is not limited to a flat plane, and hence, deformation forms a pyramid or cone structure, as shown in Figure 37 [221].

**Figure 38:**
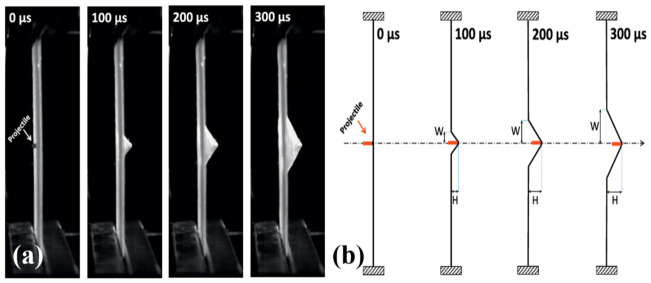
Formation of a pyramid: (**a**) visualization of a high-speed camera and (**b**) schematization (reprinted with permission from [223], Elsevier, 2013).

The visual study of the deformation pyramid was conducted by Ha-Minh et al. [223] in relation to the time recorded by a high-speed camera, as depicted in Figure 38a,b [223]. They further pointed out that the deformation pyramid evolves according to the width (W) and height (H), affecting the overall structural integrity of the composite.

The deflection response on single-layer Spectra 900/vinyl ester resin and polyurethane laminate was investigated by Lee et al. [224]. The study confirmed that the deviation in both laminates is in a range of 9 to 12 mm under static penetration loading at 0.000254 m/s before failure.

### 8.4. Bowing

**Figure 39:**
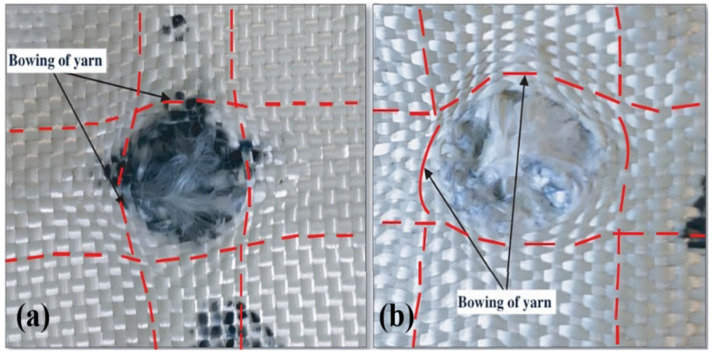
The bowing of yarn: (**a**) 2D plain weave and (**b**) 3D warp interlock panel fabric (adopted from [225], Sage, 2019).

Bowing is described as the condition when warp yarns deviate angularly from the weft yarns. Bowing occurs in fabric either by the passage of the projectile in fabric or by a stress wave spreading from the impacted region [122]. Further, Abtew et al. [225] defined bowing as transverse fabric deformation while investigating the 2D plain weave and 3D warp interlock fabrics, as illustrated in Figure 39 [225]. They observed that the bowing effect at the impact location was more significant in 3D warp interlock in contrast to 2D plain weave fabric due to the stiffer and more stable weave architecture of the 2D fabric. Moreover, the bowing in 3D fabric was due to the pushing effect of the projectile during impact, whereas in 2D fabric, bowing occurs due to stress waves traveling through crossover points.

The bowing phenomenon has a direct impact on the ballistic response of the fabric. Two-dimensional plain weave fabric exhibits less bowing due to a stable structure, resulting in a better distribution of stress waves and enhanced energy absorption. In contrast, 3D warp interlock fabric shows greater bowing, which leads to localized deformation and reduced impact energy dissipation. Hence, with excessive bowing, the fabric bends and deforms at the impacted area when subjected to projectile loads. The impact becomes concentrated in a smaller area instead of spreading across the fabric, resulting in localized damage and reduced protective performance.

## 9. Conclusions and Future Scope

Advances in materials and fabric architectures have significantly improved the performance of soft body armor against low-velocity ballistic threats. High-strength, high-modulus fibers enable effective stress transfer and energy dissipation, with the fabric structure and yarn-level parameters playing a decisive role in impact resistance. Plain weave fabrics exhibit a superior ballistic performance due to strong interlacing, while hybrid material systems offer synergistic benefits for lightweight armor design. Projectile geometry and impact conditions strongly influence penetration behavior, highlighting the importance of standardized testing protocols and back-face signature evaluation to assess trauma risk. Performance enhancement strategies, including surface modification, latex impregnation, three-dimensional fabric architectures, and shear thickening fluid incorporation, have demonstrated notable improvements in impact resistance. The integration of shear thickening fluids with natural fibers further supports sustainable and flexible armor development. Overall, these insights provide a foundation for designing next-generation soft body armor systems with improved protection and functionality.

Future work should focus on developing fully integrated, multi-scale design strategies to optimize hybrid systems, such as combining synthetic fibers with bio-derived reinforcements, to achieve a balance between sustainability, cost, and ballistic efficiency. In addition, attention should be given to ensuring durability under varying environmental conditions, including moisture, temperature, and UV exposure. Finally, data-driven approaches, including machine learning techniques for material selection and structural optimization, are needed to enhance the multifunctionality and real-world applicability of body armor systems.

## Figures and Tables

**Table 1:** Properties of aramid fibers [49,50].

Fiber Type	Density	Tensile Modulus	Tensile Strength	Fracture Strain
	g/cm^3^	GPa	MPa	%
Kevlar 29	1.44	70	3300	4.2
Kevlar 49	1.45	135	3300	2.8
Kevlar 129	1.45	99	3400	3.3
Kevlar 149	1.47	143	3600	1.5
Twaron	1.45	121	3100	2.0

**Table 2:** Properties of UHMWPE fibers [2].

Fiber Type	Density	Tensile Modulus	Tensile Strength	Fracture Strain
	g/cm^3^	GPa	MPa	%
Spectra 900	0.97	73	2400	2.8
Spectra 1000	0.97	103	2830	2.8
Spectra 2000	0.97	124	3340	3.0
Dyneema	0.97	87	2600	3.5

**Table 3:** Physical and mechanical properties of glass fibers [54].

Fiber Type	Density	Tensile Modulus	Tensile Strength	Fracture Strain
	g/cm^3^	GPa	MPa	%
E-glass	2.58	72	2600	3.0
S-glass	2.48	90	4400	5.7

**Table 4:** Physical and mechanical properties of carbon fibers (PAN) [27].

Fiber Type	Density	Tensile Modulus	Tensile Strength	Fracture Strain
	g/cm^3^	GPa	MPa	%
Celion	1.80	230	4000	1.8
Aksaca	1.78	240	4200	1.8

**Table 5:** Physical and mechanical properties of ceramic fibers [17].

Fiber Type	Density	Tensile Modulus	Tensile Strength	Fracture Strain
	g/cm^3^	GPa	MPa	%
Alumina	2.50	152	1720	2.0
Silicon carbide	2.80	420	4000	0.6

**Table 6:** Properties of Zylon fibers [58].

Fiber Type	Density	Tensile Modulus	Tensile Strength	Fracture Strain
	g/cm^3^	GPa	MPa	%
Zylon	1.56	270	5800	2.5

**Table 7:** Evolution in the nomenclature of 3D fabric.

S.N.	Former Name	Updated Name
1	Orthogonal 3D woven fabric	Orthogonal interlock, through-thickness
2	3D warp interlock	Orthogonal interlock, layer-to-layer
		Angle interlock, layer-to-layer
3	3D angle interlock	Angle interlock, through-thickness

**Table 8:** Ballistic testing standards NIJ-0101.06.

Level	Round	Caliber	Ammunition Type	Mass (g)	Minimum Velocity (m/s)	Maximum BFS (mm)	Shoot per Panel
IIA	1	9 mm	FMJ	8	373	44	6
	2	0.40	S&W FMJ	11.7	352	44	6
II	1	9 mm	FMJ RN	8	398	44	6
	2	0.357 Magnum	JSP	10.2	436	44	6
IIIA	1	0.357 SIG	FMJ FN	8.1	448	44	6
	2	0.44 Magnum	SJHP	15.6	436	44	6
III	1	7.62 mm	FMJ	9.6	847	44	6
IV	1	0.30 Caliber	M2AP	10.8	878	44	1 or 6

FMJ: Full Metal Jacket, JSP: Jacketed Soft Point, SJHP: Semi-Jacketed Hollow Point.

**Table 9:** HOSDB ballistic resistance protection levels.

Protection Level	Description
**HG1/A**	This is the lowest ballistic protection level for HOSDB, and the BFS can be up to 44 mm and cannot exceed this.
**HG1**	This is recommended for use in low-risk areas and can be operated overtly and covertly.
**HG2**	Recommended for special operations where the chance of shootings is high.
**HG3**	Suggested for heavy-duty body armor and generally employed with RF and SG hard armor plates.
**SG1**	Provides protection against shotguns at close range.
**RF1**	Ensures defense against soft-core ammunition from rifles.
**RF2**	Safeguards against steel core ammunition from rifles and the maximum protection for hard armor panels.

**Table 10:** Stab-resistant protection-level strike energies.

Protection Level	Energy Level	Strike Energy (J)	Over Test Energy (J)
**Level 1**	Low	24	36
**Level 2**	Medium	33	50
**Level 3**	High	43	65

## Data Availability

Not applicable.

## Abbreviations

BFS	Back-face signature
B_4_C	Boron carbide
CFRP	Carbon fiber-reinforced polymer
CNT	Carbon nanotube
*µ*	Coefficient of friction
CSR	Critical shear rate
*ε*	Failure strain
FESEM	Field emission scanning electron microscopy
FEA	Finite element analysis
GO	Graphene oxide
µm	Micrometer
MWCNT	Multi-walled carbon nanotube
NIJ	National Institute of Justice
ODT	Order–disorder transition
PBO	Poly(p-phenylene-2,6-benzobisoxazole)
PAN	Polyacrylonitrile
PEG	Polyethylene glycol
PMMA	Poly-methyl-methacrylate
PSt-EA	Polystyrene ethyl acrylate
KOH	Potassium hydroxide
pH	Potential of hydrogen
SEM	Scanning electron microscopy
$\dot{\gamma }$	Shear rate
STF	Shear thickening fluid
SiO_2_	Silica dioxide
SiC	Silicon carbide
SiCN	Silicon carbonitride
NaOH	Sodium hydroxide
SATNAG-2920	Standardization Agreement
σ	Stress
3D	Three-dimensional
TiO_2_	Titanium dioxide
2D	Two-dimensional
HOSDB	UK Home Office Scientific Development Branch
UHMWP	Ultra-high-molecular-weight polyethylene
USD	United States dollar
UTM	Universal testing machine
η	Viscosity
*ϕ*	Volume fraction
wt.%	Weight percent
XRD	X-ray diffraction
ZnO	Zinc oxide
